# Enabling large-scale genome editing at repetitive elements by reducing DNA nicking

**DOI:** 10.1093/nar/gkaa239

**Published:** 2020-04-21

**Authors:** Cory J Smith, Oscar Castanon, Khaled Said, Verena Volf, Parastoo Khoshakhlagh, Amanda Hornick, Raphael Ferreira, Chun-Ting Wu, Marc Güell, Shilpa Garg, Alex H M Ng, Hannu Myllykallio, George M Church

**Affiliations:** 1 Department of Genetics, Harvard Medical School, Boston, MA, 02115 USA; 2 Wyss Institute for Biologically Inspired Engineering, Boston, MA, 02115 USA; 3 LOB, Ecole Polytechnique, CNRS, INSERM, Institut Polytechnique de Paris, 91128 Palaiseau, France; 4 John A. Paulson School of Engineering and Applied Sciences, Harvard University, Cambridge, MA, 02138 USA; 5 Department of Biology and Biological Engineering, Chalmers University of Technology, SE412 96 Gothenburg, Sweden; 6 Pompeu Fabra University, Barcelona Biomedical Research Park, 08003 Barcelona, Spain

## Abstract

To extend the frontier of genome editing and enable editing of repetitive elements of mammalian genomes, we made use of a set of dead-Cas9 base editor (dBE) variants that allow editing at tens of thousands of loci per cell by overcoming the cell death associated with DNA double-strand breaks and single-strand breaks. We used a set of gRNAs targeting repetitive elements—ranging in target copy number from about 32 to 161 000 per cell. dBEs enabled survival after large-scale base editing, allowing targeted mutations at up to ∼13 200 and ∼12 200 loci in 293T and human induced pluripotent stem cells (hiPSCs), respectively, three orders of magnitude greater than previously recorded. These dBEs can overcome current on-target mutation and toxicity barriers that prevent cell survival after large-scale genome engineering.

## INTRODUCTION

Endogenous transposable elements (TEs) such as Alu (1), long interspersed elements-1 (LINE-1) (2–4) or human endogenous retro viruses (HERV)(5) make up ∼45% of the human genome (5). While originally characterized as ‘junk DNA,’ TEs are now recognized as having shaped the evolution of the human genome, and their residual transposition activity has been linked to human physiology and disease. For instance, LINE-1 sequences (17% of the genome) are highly active in certain somatic cells (6), can disrupt gene expression (4) and are suspected of having roles in human diseases (2–3,7–8) and aging (9,10). Alu and HERV have been associated with aging (11) and multiple sclerosis (12,13), respectively. The most direct test of such hypotheses would involve genomically inactivating these elements, but this has been effectively out of reach because it would require editing large numbers of distinct loci, challenging the capacity of current editing methods and the ability of cells to tolerate their activity due to the high toxicity of double-strand DNA breaks (DSBs) (14,15). The current record for simultaneous inactivation of TEs—62 elements—was achieved using CRISPR/Cas9 (16) on porcine endogenous retroviruses (PERVs) in a transformed pig cell line. Two years later a live pig was born with genome-wide KO of all 25 PERVs (17).

CRISPR/Cas9 incurs toxicity because it generates double-strand DNA breaks (DSBs) (14). These DSBs contribute to its high genome-editing efficiency by potently triggering endogenous processes that repair them with non-random (18,19) or user-specified variations, but high numbers of concurrent DSBs overwhelm these processes and cause cell death. Recently, however, two types of CRISPR/Cas9 ‘base editors’ (BEs) were developed (Supplementary Table S1) by fusing variants of Cas9 that are either ‘dead’ (dCas9; both nuclease domains inactivated) or ‘nicking’ (nCas9; one nuclease domain inactivated), in which the DSB-generating nuclease domains are disabled, to a nucleotide deaminase. Cytidine base editors (CBEs: either dCBEs or nCBEs (20)) employ cytidine deaminases and convert C:G base pairs to T:A, while adenine base editors (ABEs: either dABEs or nABEs (21)) use adenine deaminases and convert A:T base pairs to G:C. Using properly designed gRNAs, C→T conversions may be used to generate stop codons to knock-out protein coding genes of interest (14). The target nucleotide must be within the editing window of base three to nine of the gRNAs to be efficiently edit. Random genome-wide off-target SNVs have been reported when using CBEs that appear to be independent of gRNA binding sites (22,23), additionally RNA off-targets have been reported when using BEs (24,25). In addition to off-target mutations, base editors will often deaminate adjacent bases to the intended target, limiting the applications of these tools to when additional base alteration is tolerated or not possible. Improvements in base editing purity—the frequency of desired base conversion within target window—have been achieved by fusing bacterial mu-gam protein to the base editor to generate nCBE4-gam (26). Naming conventions for all BEs are summarized in Supplementary Table S2.

To achieve similar efficiencies to native Cas9 all base editor generations beyond the first are nBEs. As a result, base editing has been broadly demonstrated with high efficiency in a range of species including human zygotes (27). A main motivation for developing BEs that avoid DSBs was to reduce the level of random versus user-specified mutations caused by ‘live’ Cas9, but the reduced toxicity of BEs accrued by avoiding DSBs has also facilitated the editing of single targets in sensitive cell types such as human induced pluripotent stem cells (hiPSCs) (28) and up to 20 copies in pigs (29). However, whether these BEs can enable concurrent editing in human cells of sites as numerous as high copy TEs has not been explored but is particularly relevant to genome wide recoding efforts such as genome project write (30) (GP-write). While single-strand breaks (SSBs) are less toxic and more readily repaired than DSBs, two adjacent nicks in complementary strands leads to DSBs (31) that are not readily repaired (32). To recode the human genome would require an estimated 4438–9811 precise modifications to remove all instances of one of the three stop codons (33), while individual delivery of thousands of gRNAs is out of scope for this manuscript; we separate the challenge of multiple gRNA delivery by using a single gRNA targeting high copy number elements to select the best available genome editing tools and determine the maximum DNA edits that can be currently tolerated.

## MATERIALS AND METHODS

### Transposable element gRNA design

gRNAs targeting Alu were designed by downloading the consensus sequence from repeatmasker (http://www.repeatmasker.org/species/hg.html). LINE-1 gRNAs were designed based on the consensus of 146 ‘Human Full-Length, Intact LINE-1 Elements’ available from the L1base 2 (34). HL1gR 1–6 were designed to generate stop codons from C→T deamination mutations. EN, RT and ENRT pairs of gRNAs were designed to create moderate size deletions (200–800 bp) easily distinguishable from their wild-type full-length forms by gel visualization. HERV-W gRNAs were designed based on the consensus sequence of the 26 sequences identified by Grandi *et al.* (35) that can lead to the translation of putative proteins.

### qPCR evaluation of copy number across repetitive element targeting gRNAs

The quantitative polymerase chain reaction (qPCR) reactions were performed using the KAPA SYBR FAST Universal 2× qPCR Master Mix (Catalog #KK4602) according to the manufacturer's instructions. The LightCycler 96 machine from Roche was used to perform the qPCRs and the results were extracted using the LightCycler 96 SW 1.1 software. The following thermocycling conditions were used: ‘preincubation’ stage = 95°C for 180 s; ‘2-step cycling’ stage: annealing = 95°C for 3 s and elongation = 60°C for 20 s; ‘Melting’ stage = keep standard. The following primers were used:

ZY-JAK2-F AGCAAGTATGATGAGCAAGC; SB-JAK2-R AAAACAGATGCTCTGAGAAAGGC; P1(b)_REBE_F TAGGAACAGCTCCGGTCTACA; P1_REBE-ilu_R AATGCCTCGCCCTGCTTCGG; P5_REBE-ilu_F CCAATACAGAGAAGTGCTTAAAGG; P5_REBE-ilu_R CTTGGAGGCTTTGCTCATTTCT; P7_REBE-ilu_F CCCATCAGTGTGCTGTATTCAGG; P7_REBE-ilu_R GGCCTTCTTTGTCTCTTTTG; P13_REBE-ilu_F AACAGGCTCTGAAATTGTGGC; P13_REBE-ilu_R GCTGGCCTCATAAAATGAGTTAG; P15_REBE-ilu_F GTTCTGGCCAGGGCAATCAG; P15_REBE-ilu_R CCTGAGACTTTGCTGAAGTTGC

### Bioinformatic alignment and copy number analysis

Fasta sequences of hg38 reference genome were downloaded from Ensembl (ftp://ftp.ensembl.org/pub/release-95/fasta/homo_sapiens/dna/). Alignment analysis of the gRNA sequences to all chromosomes was performed using the R library Biostrings v2.40.2 and plotted using the R library ggplot2 3.3.0.

### SpCas9 and gRNA plasmids used for genome editing

The following Cas9 plasmids were used: pCas9_GFP (Addgene #44719), hCas9 (Addgene #41815). Base editing plasmids used: pCMV_BE3 (Addgene #73021), pCMV_BE4 (Addgene #100802), pCMV_BE4-gam (Addgene #100806), ABE 7.10 (Addgene #102909). The gRNAs used in this study were synthesized and cloned as previously described (36). Briefly, two 24mer oligos with sticky ends compatible for ligation were synthesized from IDT for cloning into the pSB700 plasmid (Addgene Plasmid #64046).

### SaCas9 and gRNA plasmids used for genome editing

Cas9 plasmid: pX600-AAV-CMV::NLS-SaCas9-NLS-3xHA-bGHpA (Addgene #61592). Base editing plasmid: SaBE4-gam (Addgene #100809). The gRNAs used in this study were synthesized and cloned as previously described (37). Briefly, two 24mer oligos with sticky ends compatible for ligation were synthesized from IDT for cloning into the BPK2660 plasmid (Addgene Plasmid #70709).

### Maintenance and transfection of HEK 293T cells

HEK293T cells were obtained from ATCC with verification of cell line identification and mycoplasma negative results. They were expanded using 10% fetal bovine serum (FBS) in high-glucose Dulbecco's modified Eagle's medium (DMEM) with glutamax passaging at a typical rate of 1:100 and maintained at 37°C with 5% CO_2_. Transfection was conducted using Lipofectamine 2000 (Thermofisher Catalogue # 11668019) using the protocol recommended by the manufacturer with slight modifications outlined below. Twenty-four hours before transfection ∼1.0 × 10^5^ cells were seeded per well in a 12-well plate along with 1 ml of media. A total of 2 μg of DNA and 2 μl of Lipofectamine 2000 were used per well. For Cas9 plasmids, the DNA content per well was 1 μg of pCas9_GFP mixed with 1 μg of gRNA-expressing plasmid. For BE plasmids, 1.5 μg of BE was mixed with 0.5 μg of gRNA plasmid. In the dBE versus nBE comparison used to generate Figure 4, Pifithrin-α (10 ng/μl) from Sigma-Aldrich P4359 (source # 063M4741V, Batch # 0000003019) and bFGF from Thermo Scientific (catalog # 13256029) was added to the media 30 min before transfection and maintained in the first day media change.

### FACS single cell direct NGS preparation

To quantify early genetic editing in cells transfected with Cas9/BE and gRNA expression plasmids, single cells were sorted and prepared as follows. Two days post-transfection, single cells were FACS-sorted into 96-well PCR plates containing 10 μl of QuickExtract™ DNA Extraction Solution (Epicentre Cat. # QE09050) per well and genomic DNA (gDNA) was extracted using the manufacturer's protocol. Briefly, the sorted plates were sealed, vortexed and heated at 65°C for 6 min then 98°C for 2 min. The NGS library was prepared as described later below.

### Single cell clonal isolation and sequence verification

Single cells were FACS-sorted into flat bottom 96-well plates containing 100 μl of DMEM with 10% FBSand 1% Penicillin/Streptomycin per well. Sorted plates were incubated for ∼14 days until well-characterized colonies were visible, with periodic media changes performed as necessary. To extract gDNA, the cells were first detached using 30 μl TrypLE™ Express (Thermofisher Cat. # 12604021) and neutralized with 30 μl growth media. Then, 4 μl of the resulting cell suspension was transferred to 10 μl of QE. Genomic DNA was extracted according to manufacturer's protocol, as described previously.

### Nested PCR Illumina MiSeq library preparation and sequencing

Library preparation was conducted as previously described (38). Briefly, genomic DNA was amplified using locus-specific primers (Supplementary Table S3) attached to part of the Illumina adapter sequence. A second round of PCR included the index sequence and the full Illumina adapter. All PCRs were carried out using KAPA HiFi HotStart ReadyMix (KAPA Biosystems KK2602) according to the manufacturer's thermocycler conditions. Libraries were purified using gel extraction (Qiagen Cat. # 28706), quantified using Nanodrop and pooled together for deep sequencing on the MiSeq using 150 paired end (PE) reads.

### NGS indel analysis

Raw Illumina sequencing data was demultiplexed using bcl2fastq. All paired end reads were aligned to the reference genome using bowtie2 (39) and the resulting alignment files were parsed for their cigar string to determine the position and size of all indels within each read using a custom perl script (https://github.com/CRISPRengineer/mutation_indel). All indels that were sequenced in both the forward and reverse reads were summed across all reads and reported for each sample along with the total number of reads. Indels within a 30 bp window from the 5′ start of the gRNA proceeding through the PAM and extending an additional seven bp's (for a 20 bp gRNA) were counted and summed for each sample.

### Dual gRNA deletion frequency NGS analysis

Reads were analyzed for dual gRNA large deletions by detecting sequences in between the gRNAs to indicate the full length unedited (at least not dual gRNA-edited) and sequences beyond the normal wild-type amplicon that only appear when the deletion has occurred to identify deletion reads. The custom perl script used for analysis is available at https://github.com/CRISPRengineer/dual_gRNA.

### NGS base editing deamination analysis

All paired end reads were aligned to the reference genome using bowtie2, and the resulting alignment files were converted to bam, sorted, indexed, and variant called using samtools (40). All SNV data within a 30 bp window from the 5′ start of the gRNA proceeding through the PAM and extending an additional seven bp's (for a 20 bp gRNA) are reported to analyze the editing window and purity of editing. The custom perl script used for analysis is available at https://github.com/CRISPRengineer/deamination_report.

### Site directed mutagenesis to remove remaining nick from base editors

We deactivated the remaining nuclease domain of Cas9 from nCBE4 (Addgene #100802), nCBE4-gam (Addgene #100806), pCMV-ABE7.10 (Addgene #102919), pCMV-AncBE4max (Addgene #112094) and pCMB_ABEmax (Addgene #112095). Agilent QuikChange XL Site-Directed Mutagenesis Kit (catalogue # 200517) was used with the following primer sequences:

SpCas9-fwd—tttatctgattacgacgtcgatgccattgtaccccaatcctttttg
SpCas9-rev—caaaaaggattggggtacaatggcatcgacgtcgtaatcagataaa

### Propidium Iodide and Annexin V staining and FACS analysis

Cells were dissociated with TrypLE, diluted in an equal volume of phosphate-buffered saline (PBS) and then centrifuged at ∼300 *g* for 5 min at room temperature. We resuspended samples into 500 μl PBS and half of the cells were pelleted for later gDNA analysis. The remainder was centrifuged and resuspended into 100 μl of Annexin V Binding Buffer (ref #V13246) diluted into ultrapure water at a 1:5 ratio. Subsequently, we added 5 μl of Alexa 647 Annexin V dye (ref #A23204) and incubated samples in the dark for 15 min. We then added 100 μl of Annexin V Binding Buffer and added 4 μl of Propidium Iodide (ref #P3566) diluted into the Annexin V Binding Buffer at a 1:10 ratio. Samples were incubated in the dark for another 15 min. Cells were washed with 500 μl of Annexin V Binding Buffer and centrifuged again to be finally resuspended into 400 μl of Annexin V Binding Buffer. All samples were filtered using a cell strainer and were run on the LSR 11 using a 70-μm nozzle. Analysis was conducted using FlowJo software.

### Karyotype analysis of LINE-1 dBE-edited 293T single cell clones

Stable HEK 293T edited isolated cell lines (nCBE4-gam, dCBE4-gam, ABE and dABE) were expanded and karyotypically compared with the control groups and the wild-type HEK 293T. Actively growing cells were passaged 1–2 days prior to sending to BWH CytoGenomics Core Laboratory. The cells were received by the core at 60–80% confluency. Chromosomal count, variances and abnormalities were investigated.

### Whole genome sequencing off-target analysis

The top 293T edited clones used for the karyotype analysis were expanded and isolated with the 293T population frozen before initial transfection (pre293T) along with a control 293T population expanded for an equivalent amount of time as the other mutant clones sequenced(post293T). DNA was extracted using the Qiagen DNeasy Blood and Tissue kit (cat-#69506) and were sequenced using Illumina PE 150 to a depth of ∼30×. Alignment and variant calling was provided by the Harvard Chan Bioinformatics Core, Harvard T.H. Chan School of Public Health, Boston, MA, USA using an analysis pipeline based on bcbio framework (https://github.com/bcbio/bcbio-nextgen). For WGS data, BWA (v0.7.17) was used to map sequencing reads to the reference human genome (hg38). We called SNPs and indels using somatic tumor-normal approach (using a control sample as a normal, and edited samples as ‘tumor’), and required 3 variant callers (vardict, v.2019.06.04, mutect2 (from gatk 4.1.2.0), strelka2, v2.9.10) to confirm a variant to be called (a similar approach was taken by Zuo *et al.* (24). In the case of RNA-seq data, we used STAR (v.2.6.1d) to align reads and RNA-seq specific gatk-based variant calling pipeline, with parameters and filters recommended by GATK best practices for RNA-seq variant calling (https://software.broadinstitute.org/gatk/documentation/article.php?id=3891), followed by filtering out variants at RNA editing sites according to the RADAR (v.2-20180202) database. We used GATK 3.8 to call variants in RNA-seq data, because our validation has shown the superior precision of gatk 3.8 over gatk 4.1.2.0 when using RNA-seq reads. Due to the variability of coverage in RNA-seq data, variants were called in a single batch and only variants called as het, hom or hom ref in all samples were considered for the downstream analysis. We filtered out variants at sites matching gRNA using bedtools (2.27.1) and a custom bash script and used R-studio and ggplot2 for the downstream analysis.

### RNA-seq analysis after base editing

293T cells were transfected with HL1gR4 and either nABE, dABE, nCBE4-gam or dCBE4-gam and cell pellets were isolated after 48 h for DNA and RNA extraction. DNA was prepared for targeted amplicon sequencing as previously described. Cells for RNA-seq were lysed with TRIZOL (ThermoFisher 15596026) and total RNA was extracted using Zymo RNA mini prep kit (Zymo R2052). RNA was quantified using Qubit Fluorometer (ThermoFisher Q10211) and RNA integrity was confirmed by presence of two ribosomal bands and absence of degraded smears by gel electrophoresis. mRNA-seq libraries were prepared using KAPA mRNA HyperPrep (KAPA KK8580) using 1 μg total RNA. Libraries were pooled and sequenced on an Illumina MiSeq.

### RNA-seq analysis of LINE-1 edited living cell lines

The RNA of 293T LINE-1 edited clones (1.37–3.4% deamination by nCas9-CBE4-gam editing) was extracted by treatment with TRIzol (ThermoFisher Scientific, cat-# 15596018) followed by Direct-zol RNA Kit (Zymo Research, cat # R2072), according to the manufacturer's instructions. All samples were prepared from biological duplicates; the parental culture was divided into two cultures and passaged once before extraction. A total of 500 ng RNA of each of the samples, as quantified by Qubit (QubitTM RNA HS Assay Kit, ThermoFisher Scientific, cat-# Q32852), was used to prepare the libraries using an NEBNext Directional RNA Library Prep Kit (New England Biolabs, cat-# E7765S) in conjunction with the Poly(A) mRNA Magnetic Isolation Module (New England Biolabs, cat-# E7490), and following the manufacturer's instructions. Deamination frequency in the RNA was analyzed using the standard deamination analysis pipeline used for genomic DNA. Read counts were generated by mapping reads to a human reference genome (GRCh38.p12, using the PRI version from www.genecodegenes.org) using STAR. Differential gene expression analysis was performed in EdgeR version 3.24.3: Lowly expressed genes with less than two counts per million in two or more samples were filtered out, the libraries were normalized using trimmed mean of M values (TMM) normalization and differentially expressed genes were identified by using the exact test on the tagwise dispersion to compare the expression of each of the clones to the control sample. The Benjamini-Hochberg method was used to adjust *P*-values for multiple testing. Multidimensional Scaling distances were generated by using the plotMDS function of EdgeR on the filtered and normalized libraries and plotted using ggplot.

### Maintenance and expansion of human iPSCs

Human iPSCs were cultured with mTeSR medium on tissue culture plates coated with Matrigel (BD Biosciences). For routine passaging, iPSCs were digested with TrypLE (Thermofisher # 12604013) for 5 min and washed with an equal volume PBS by centrifugation at 300 *g* for 5 min. Digested iPSC pellets were physically broken down to form a single cell suspension and then plated onto Matrigel-coated plates at a density of 3 × 10^4^ per cm^2^ with mTeSR™ medium supplemented with 10 μM Y-27632 ROCK inhibitor (R_i_) (Millipore, 688001) for the first 24 h.

### Nucleofection in PGP-1 iPSCs

Thirty minutes prior to transfection media was changed to mTeSR™ supplemented with Pifithrin-α (10 ng/μl) from Sigma-Aldrich P4359 (source # 063M4741V, Batch # 0000003019); a notable spiky edge colony morphology was observed similar to when R_i_ is added. Human iPSCs were digested with TrypLE for 5 min and the single cells were washed once with PBS. (CS: 4 × 10^6^, PK: 1 × 10^6^) iPSCs were then re-suspended in 100 μl of P3 Primary Cell Solution (Lonza) supplemented with (CS: 13.5 μg, PK: 6.75 μg) of dABE plasmid, (CS: 4.5 μg, PK: 2.25 μg) of gRNA plasmid, and (CS: 2 μg, PK: 1 μg) of pMax. The combined cells and DNA were then nucleofected in 4D-Nucleofector (Lonza) using the hES H9 program (CB150). The nucleofected iPSCs were then plated onto a single well of a 6-well Matrigel-coated plate in mTeSR medium supplemented with 10 μM R_i_ and Pifithrin-α (10 ng/μl).

### Clonal isolation of PGP-1 iPSCs

96-well plates were coated with Matrigel (BD Biosciences) at a concentration of 50 μl/well. A cloning medium solution of 10% CloneR™ (StemCell Technologies #05888) and Pifithrin- α (10 ng/μl) in mTeSR™ was prepared and added to the coated wells. Cells were digested using TrypLE, which was neutralized by an equal amount of cloning medium. The cell solution was then centrifuged at 300 × *g* for 5 min, the supernatant was aspirated, and the cell pellet was resuspended in the cloning medium. The cells were then passed through a 40-μm cell strainer and were FACS-sorted into (i) individual wells containing warm cloning medium at a density of 1 cell/well and (ii) 2 × 96-well PCR plates for direct NGS analysis. To prevent disturbance, there was no media change during the first 48 h, and the plates were not removed from the incubator during this period. A half-medium change was performed on days 3 and 4 with cloning medium. The growing colonies were monitored and a mTeSR™ medium change was done daily for the following days until extracting the DNA using QuickExtract™ and proceeding with library preparation and sequencing.

### Genomic integration and long-term LINE-1 editing in PGP-1 iPSCs

The base editor constructs were cloned into the piggybac dox inducible expression vector PB-TRE-dCas9-VPR (Addgene #63800) including a puromycin selection marker. HL1gR4 was cloned into the PB-EF1α-MCS-IRES-Neo PiggyBac cDNA Cloning and Expression Vector (catalog #: PB533A-2) under a constitutive U6 promoter. A total of 2 μg of piggybac base editor plasmids were transfected with 8 μg of super transposase using the nucleofection conditions described previously. Cells were selected for puromycin (1μg/ml) for 10 days. Cells were then transfected with 2 μg PB-gRNA-HL1gR4 and 8 μg of super transposase then selected for G418 resistance for 12 days. Doxycycline (1 μg/ml) was added at day 12 to induce expression of the base editors and begin editing at LINE-1 for 21 days. Genomic DNA was isolated and analyzed for LINE-1 editing over the time course then single cell isolation was performed as previously described.

### Statistical analysis

Statistical analysis was conducted using the student's *t*-test. Differences were considered significant if P-value was <0.05. * −0.01 < *P* < 0.05, ** −0.001 < *P* < 0.01, *** − *P* < 0.001, **** −*P* < 0.0001.

## RESULTS

### gRNA design and copy number estimation of transposable elements

**Figure 1.**
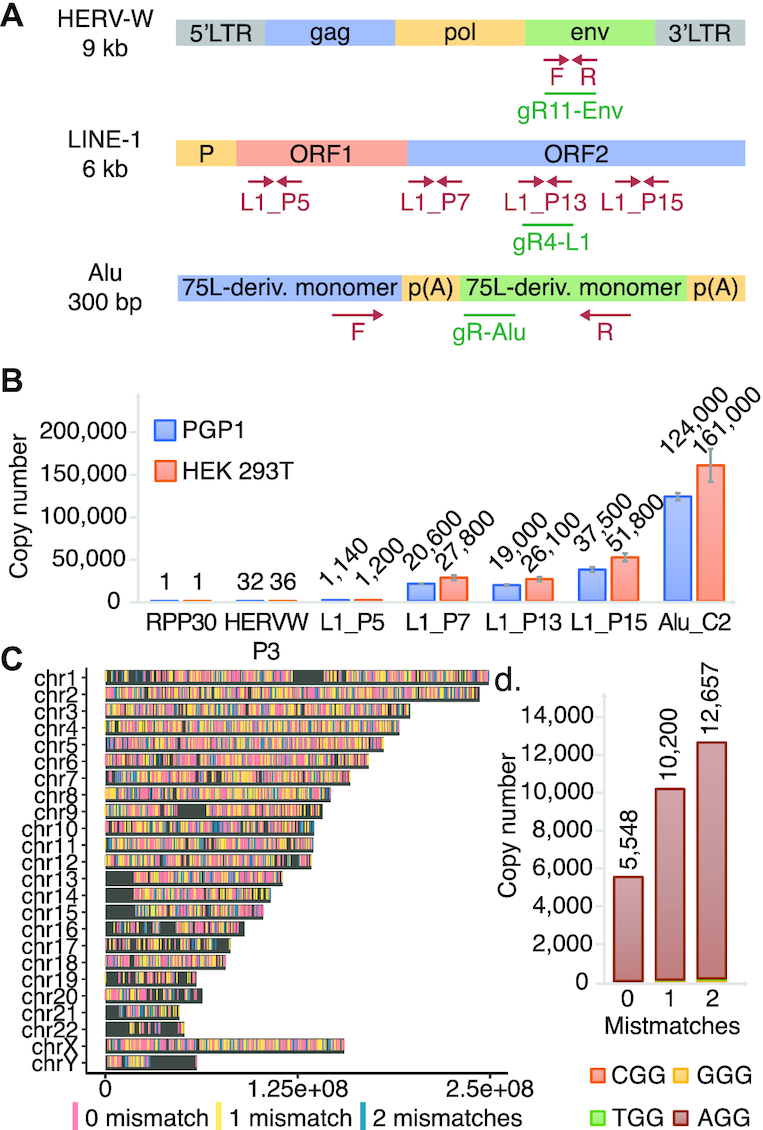
Utilizing high copy repetitive elements for the development of less toxic DNA editors. (**A**) A summary of HERV, LINE-1 and Alu. Representation of TEs with qPCR primer sites shown in red and gRNAs shown in green. (**B**) qPCR estimation of LINE-1 copy number per haploid genome across the element compared to single copy number controls in PGP1 and HEK 293T. Errors bars display standard deviation, *n* = 3. (**C**) Genome wide distribution of HL1gR4. (**D**) HL1gR4 copy number and PAM distribution.

To assess the efficiency and toxicity of current editing technologies as applied to TEs, we designed and tested gRNAs against Alu, LINE-1 and HERV which vary in copy number from 30 to >100 000 across the genome (Figure 1A). Alu and LINE-1 gRNAs were respectively designed on the consensus sequences obtained from repeatmasker (41) (Supplementary Table S4) and on the consensus of 146 full-length sequences that encodes both functional ORF1 and ORF2 proteins (34). At last, gRNAs against HERV-W, one subfamily of HERV, were designed on the consensus of putatively active retro-viruses (35) (Supplementary Table S4). We performed qPCRs of genomic DNA (gDNA) generated using consensus sequence-based primers to estimate the relative abundance in HEK 293T and PGP1 iPSCs (Figure 1A). The copy number of HERV-W, LINE-1 and Alu elements at the edited sites were respectively estimated at 36, 26 000 and 161 000 in HEK 293T; and 32, 19 000 and 124 000 in PGP1 iPSCs (Figure 1B). The TE’s copy number in HEK 293T is higher than that in PGP1 since the former cells are largely triploid. We used a complementary bioinformatic approach as a second estimate of TE abundance by aligning our designed gRNAs to the human reference genome (Figure 1C and Supplementary Figure S1). An example of gRNA HL1gR4 targeting LINE-1 ORF2 is shown in Figure 1C. The total number of matches for HL1gR4 allowing 2 bp mismatches is 12 657, about half of our qPCR estimate, with the vast majority having an intact PAM (Figure 1D). Since the reference sequence likely undercounts TEs because of the well-known problems of assembling, aligning and mapping these sequences (42), we base our editing numbers upon the qPCR copy number estimate and editing efficiency.

### High copy-number CRISPR/Cas9 editing induces cellular toxicity and inhibits survival of edited cells

**Figure 2.**
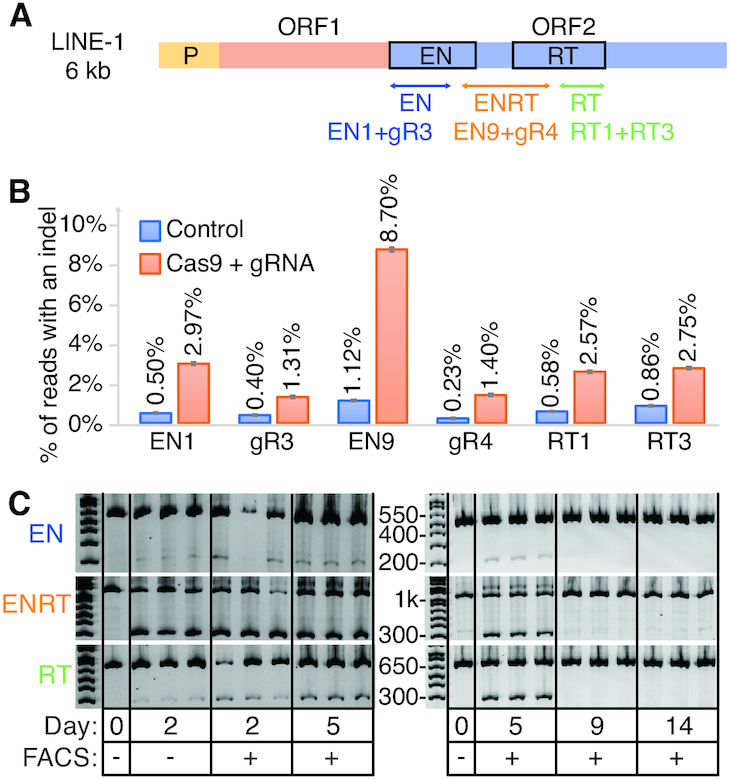
CRISPR-Cas9 based genome editing at high copy number repetitive elements is detectable but ultimately lethal. (**A**) Schematic of LINE-1 including the two protein coding genes ORF-1 and ORF-2. Three dual gRNA deletions were designed to disrupt the EN and RT domains of ORF-2. (**B**) LINE-1 gRNAs transfected with Cas9 in 293T. Displayed are single transfections with 95% confidence intervals for a proportion as the error bars. (**C**) Gel image visualizing dual gRNA deletion bands compared to wild-type control bands.

We transfected HEK 293T cells with plasmids expressing pCas9_GFP and LINE-1 targeting gRNAs to disrupt the two key enzymatic domains of ORF-2: endonuclease (EN) and reverse transcriptase (RT) (Figure 2A and Supplementary Table S4). Three days after transfection, we observed indel frequencies at the LINE-1 expected targets ranging from 1.3 to 8.7%, corresponding to an average of respectively, 339 and 2271 edits per haploid genome in the population (Figure 2B). In accord with previous reports that this degree of genetic alteration is toxic, we confirmed ∼7-fold increases in cell death and apoptosis through Propidium Iodide and Annexin V staining (Supplementary Figure S2). A follow-up time-course experiment provided evidence that cells that undergo editing at hundreds of loci do not survive. Here we transfected pairs of LINE-1 gRNAs targeting the EN, RT or both (ENRT) domains. Using pairs of gRNAs causes large deletions (∼170–800 bp) that can be detected through gel visualization (Supplementary Figure S3A). While samples from day two through five show clear editing with the expected deletion band sizes (Figure 2C), they were no longer detectable at days 9 and 14 indicating that mutated cells either died out as suggested by our previous cell death assay or were overgrown by wild-type cells. Deep sequencing of expected dual gRNA deletion bands confirmed the LINE-1 gRNA breakpoints (Supplementary Figure S3B). While there were no visible bands at day 9 and 14, we repeated this experiment and attempted to isolate clones. After early indications of editing no clones had detectable mutations at day 12 and beyond (data not shown) suggesting that any significant level of indel activity at LINE-1 is toxic or limits growth and clonal isolation. Single cell analysis confirmed the bimodal editing frequency (16) with a mean deletion frequency of 47.1% (Supplementary Figure S4).

### nCBE and nABE enable isolation of stable cell lines with hundreds of edits

With the thought that nBEs could help improve the viability of LINE-1 edited cells, we designed and tested LINE-1 targeting gRNAs (HL1gR1-6 [Supplementary Table S4]) that generate a STOP codon early in ORF-2 using C→T deamination. When we transfected HEK 293T cells with nCBE3 and each of these gRNAs, we observed levels of deamination at each target locus that, although small (∼1.07–3.91%) exceeded levels in mock transfected control cells (Supplementary Figure S5). These same CBE gRNAs could also be used with ABEs as they contain at least one adenine within their deamination window. Above control levels of base editing were observed in genomic DNA in 4/5 gRNAs for both nCBE (Supplementary Figure S5B) and nABE (Supplementary Figure S5C). While nABE with HL1gR6 exhibited the highest editing efficiency (4.94% or ∼1290 loci) three days after transfection, we used HL1gR4 going forward because it had the highest signal-to-background ratio among the more efficient gRNAs. The HL1gR4 target window also contained three efficiently coedited C’s, thus offering a clear signal of directed mutation.

**Figure 3.**
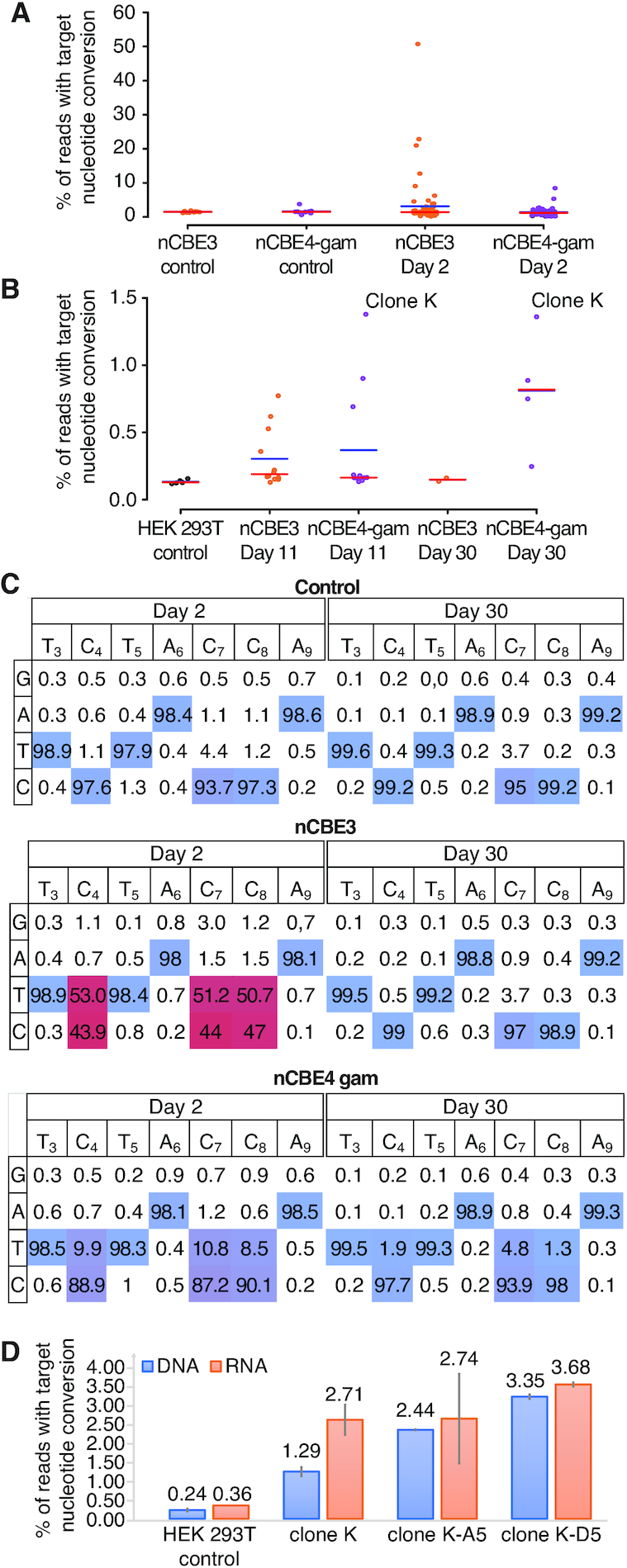
nBEs targeting LINE-1 enable survival of stable cell lines with hundreds of edits. (**A**) Base editing in HEK 293Ts two days after transfection comparing nCBE3 versus nCBE4-gam. FACS single cells (*n* = 72) are plotted as individual points representing targeted base editing nucleotide deamination. Red line indicates the median and the blue line the mean. (**B**) Single cell live culture growth and stable cell line generation at day 11 and 30. (**C**) Base editing activity across the CBE target window of ∼3–9. Comparing day 2 and 30 for analysis of initial editing activity in the most highly edited single cell for each condition. Editing efficiency is color coded from non-edited (blue) to highest editing efficiency (red). (**D**) LINE-1 deamination analyzed from either RNA or genomic DNA. SEM’s are displayed as error bars, *n* = 2.

293Ts were transfected with HL1gR4 and either nCBE3 or nCBE4-gam with control samples receiving a non-targeting gRNA. Two days post-transfection, single cells displayed an average editing of 1.41% for nCBE4-gam and 3.12% for nCBE3. While single cells were observed with up to 53.9% C→T deamination, or an estimated 14 000 loci (Figure 3A), in the highest edited single cell. nCBE3 had slightly higher mean deamination frequency than nCBE4-gam at this early timepoint but could not form any stable clonal population to the day 30 timepoint, suggesting that nCBE4-gam increased overall cell viability more than nCBE3 when targeting high copy repeats. Four surviving cell lines were isolated with deamination frequencies up to ∼1.37% of LINE-1 or an estimated ∼356 sites (Figure 3B). Data presented in Figure 3C shows both the purity of the desired deamination products and the editing window. Clone K was the highest edited stable clonal population and its targeted C→T mutation frequency from day 11 to 30 was confirmed.

By subjecting the top edited single cell isolate Clone K to another round of nCBE4-gam editing (Supplementary Figure S6A) we detected cells with up to 36.26% C→ T nucleotide conversion on day 2 (Supplementary Figure S6B), and four living clones were isolated with mutation frequencies ranging from 2.43 to 5.04%—corresponding to about 643–1315 edits (Supplementary Figure S6C). While the clone with the highest number of deaminated sites did not grow after a freezing and thawing cycle, the three other cell lines were stable in culture for a period longer than 30 days, and were termed ‘Clone K-A5’, ‘Clone K-A2’ and ‘Clone K-D5’, with respectively 643, 749 and 781 edits, respectively. This observation of the highest edited clone dying off after initial detection was observed for all types of editors. We confirmed nBE activity at the lower copy number target HERV-W with up to 9.6% average nucleotide conversion at the population level (Supplementary Figure S7). Due to the difficulty amplifying and analyzing the Alu target, likely because of high subfamily polymorphism and short repeat sequence (290bp) we proceeded exclusively with LINE-1 targeting gRNAs for the rest of the study. This higher than normal background polymorphism was also observed at LINE-1 and HERV-W but much less than with Alu.

To confirm that LINE-1 editing at the genome level was reflected on the corresponding transcripts we performed RNA-seq on Clone K, Clone K-D5 and Clone K-A5 and analyzed the percentage of C→T conversion resulting in a stop codon in ORF2 in the RNA reads (Figure 3D and Supplementary Figure S8). Theoretically, since most of the active LINE-1 subsets should generate transcripts, the presence of the expected stop codon at the messenger RNA level may indicate their inactivation. The results showed that a higher number of edits in the clones was correlated with a higher number of stop codons at the RNA level, suggesting that transcriptionally active LINE-1 subfamilies were impacted by the multiplexed editing. In Supplementary Figure S8B the number of RNA reads obtained through the standard deamination analysis pipeline, averaged over the 20 nt protospacer sequence and normalized the read counts by dividing by the size of their respective libraries, are displayed. The numbers of up and down regulated genes are found in Supplementary Figure S8C. Multidimensional scaling of the gene expression data (Supplementary Figure S8D), where the distance between the samples corresponds to leading log-fold-changes between the RNA samples, shows a clear separation between the wild-type and the three edited samples. While differences in gene expression were observed, the low level of total LINE-1 editing in these clones prevents us from concluding that LINE-1 knock-out is responsible for these changes.

### Nick-less dBEs enable the isolation of stable cell lines harboring up to 13 200 edits

Suspecting that generating single-stranded nicks genome-wide could lead to cytotoxicity, we decided to inactivate the remaining HNH nuclease domain of nCas9 by an H840A mutation in the nCas9 backbone and created a set of dCas9-BEs including dCas9-CBE4-gam (dCBE4-gam), dCas9-CBE4 (dCBE4) and dCas9-ABE (dABE). Nick-less dCas9-BEs were tested on single-locus targets to confirm their deamination activity and compare them to their nBE equivalents and the existing dCas9-CBE2 (dCBE2). dCBE4 and dCBE4-gam showed a 2.38- and 2.29-fold improvement in editing efficiency over CBE2 in 293Ts at day 5, respectively (Supplementary Figure S9). Compared to their nicking counterparts this was a 34.7 or 53.2% reduction in efficiency, but indel activity was reduced to background levels (Supplementary Figure S9A). dABE retained 40.2% of nABE’s efficiency at a single locus target while reducing indel levels to background (Supplementary Figure S9B).

**Figure 4.**
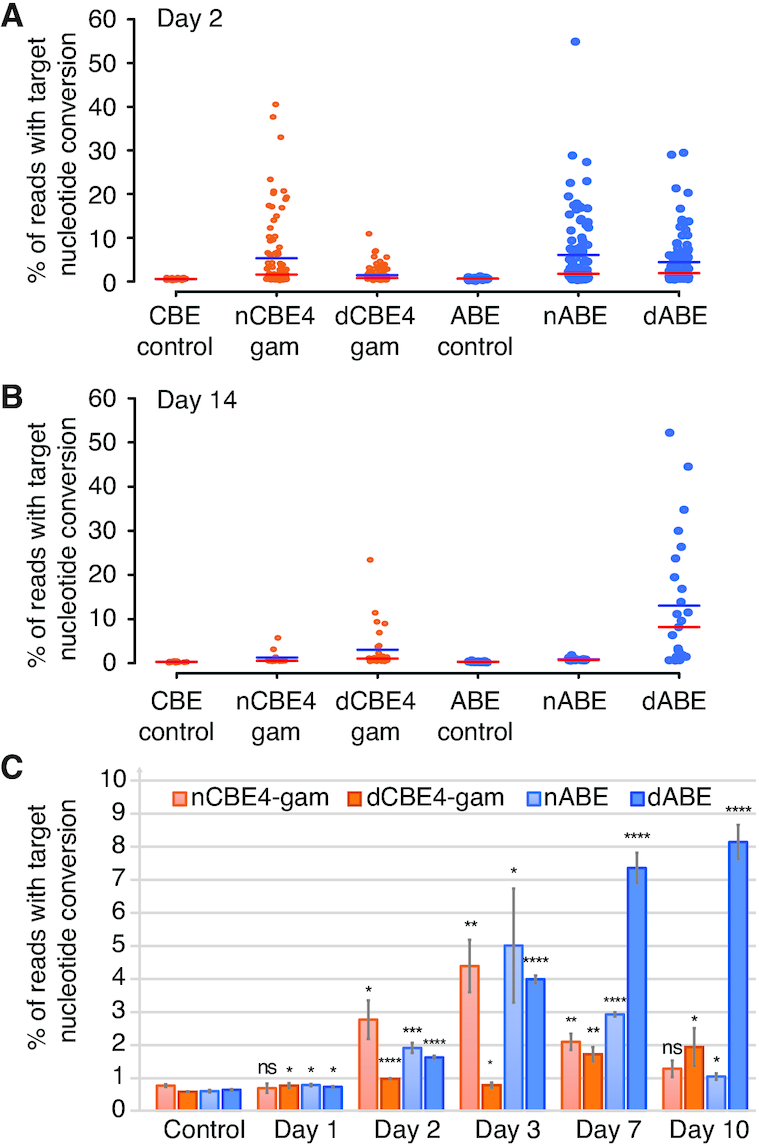
dBEs improve survival of highly edited cells with thousands of edits genome wide. (**A**) nBE compared to dBE in 293T single cells each represented as a single data point. Base editing is displayed as either target C→T or A→G conversion for CBE and ABE, respectively. The red line indicates the median and the blue line the mean. (**B**) Live single cell analysis at day 14 of the same experiment. (**C**) Deamination frequency over time comparing dBE to nBE from day one to ten. Error bars represent SEM of *n* = 3, ns (not significant), *P* ≥ 0.05; *P < 0.05; **P < 0.01; ***P < 0.001; ****P < 0.0001; by two-tailed Student's *t*-test compared to controls.

We then transfected 293T cells with HL1gR4 and either nCBE4-gam, dCBE4-gam, nABE or dABE and individually sorted and analyzed the cells for target nucleotide conversion after 2 days. Single edited cells resulted in an average editing efficiency of 5.31, 1.45, 6.08, 4.43% target nucleotide conversion for nCBE4-gam, dCBE4-gam, nABE and dABE, respectively. The top edited single cell had up to 54.9% deamination with nABE, or 14 300 loci, while we observed significant reductions to mean target nucleotide mutation frequency with dCBE and dABE when compared to their nBE equivalents (Figure 4A). In parallel, single cells were grown to determine whether viable highly edited clones could be isolated. The editing efficiency trend reversed in live cells: dBEs showed a significantly increased deamination frequency over nBEs (Figure 4B). Remarkably, dABE produced the highest edited viable clone with 50.61% targeted nucleotide conversion or an estimated 13 200 loci. We estimate that, in our highest dCBE4-gam edited clone, we have inactivated 6292 of 26 000 loci or 24.2% LINE-1 sequences. Base editors that retain nicking activity only generated a few rare cells with an editing frequency consistent with our prior experiments in Figure 3B. Results were replicated using another LINE-1 targeting gRNA and similar trends were observed (Supplementary Figure S10). The isolation of living clones with greater than 6000 edits demonstrate the required number of stable DNA modifications needed to achieve whole genome recoding can be altered within a single transient transfection. While we are only using a single repetitive element targeting gRNA true recoding efforts will require thousands of unique guides. Combined with future improvements in gRNA delivery this data indicates that such recoding efforts in mammalian cells are practical in terms of DNA toxicity.

The nucleotide composition of all bases in the gRNA and PAM are displayed for the highest edited clone and parental 293T control for each BE condition used, showing very low non-specific nucleotide conversions for both nBEs and dBEs at LINE-1 (Supplementary Figure S11). The mean single cell mutation frequency was reduced from 5.32% using nABE to 1.45% using dABE, indicating that disabling nicking resulted in a 3.67-fold decrease in editing efficiency at the day 2 timepoint (Figure 4B). Fourteen days after transfection, dBEs gained a marked advantage as compared to nBEs in the total number of viable cells, and mutation frequency of single cells. There was a 14.8-fold increase in mean editing frequency among surviving live clones when using dABE compared to nABE (Figure 4B), and a 2.38-fold increase was observed for dCBE4-gam compared to nCBE4-gam. High base editing purity was observed for both ABEs, while CBEs generated non-intended bases at the target position. dCBEs significantly reduced the generation of such non-intended bases at the target position, in particular dCBE4-gam (Supplementary Figures S9C and D). No non-specific nucleotide conversion within the gRNA was detected when targeting LINE-1 (Supplementary Figures S12). During the first three days of editing the dBEs had lower editing frequency when compared to nBEs but from day seven, dABE gained a significant edge over nABE (Figure 4C).

As a proof of concept toward the delivery of multiple individual gRNAs we transfected cells with pools of up to nine single locus targeting gRNAs to compare dABEmax versus nABEmax (Supplementary Figure S13A). Absolute editing efficiencies of up to 87.7% were observed when using nABEmax (Supplementary Figure S13B). Combinations of three, six or nine gRNAs were co-transfected and compared to their individual transfection efficiencies resulting in no significant difference observed between nicking and dead versions at this low level of multiplexing (Supplementary Figure S13C). When six gRNAs were co-delivered there was a 78.9 and 78.5% of single gRNA delivery efficiency for dABEmax and nABEmax, respectively.

HL1gR4 PCR products were analyzed to determine that only 64.1% of reads had a perfect match for the gRNA, 18.4% had a 1 bp mismatch, 3.2% with two mismatches and 13% with more than nine mismatches (Supplementary Table S5), thus most similar off-targets are actually within the LINE-1 locus. To search for random genome wide deamination off-target analysis was conducted using whole genome sequencing and RNA-seq. As previously reported (22,23), we identified genome wide off-target variants enriched for C*G→T*A mutations after CBE editing, with dCBE4-gam at 41.4% above ∼30% for the unedited samples (Supplementary Figure S14). We screened for mutations in p53 and apoptosis genes that may explain the survival advantage of highly edited cells but did not find any obviously deleterious variants (frameshift, splice site, stop codon). We also detected off-target deamination at the RNA level at day 2 (Supplementary Figure S15A-B). No long-term effects of RNA mutation spectrum were observed in the stable CBE edited clones after 30–70 days (Supplementary Figure S15C). Chromosomal integrity analysis was performed for clones edited at LINE-1 with nABE, dABE, nCBE4-gam and dCBE4-gam. The karyotype results are shown in Supplementary Table S6 and show that the top edited clones are not significantly different from control groups in terms of the total number of aberrations (Supplementary Figures S16 and 17). Further analysis in a karyotypically normal and stable cell line is required to fully assess chromosomal stability after large-scale genome editing.

### dABE allows the isolation of hiPSCs harboring up to 12 200 edits

**Figure 5.**
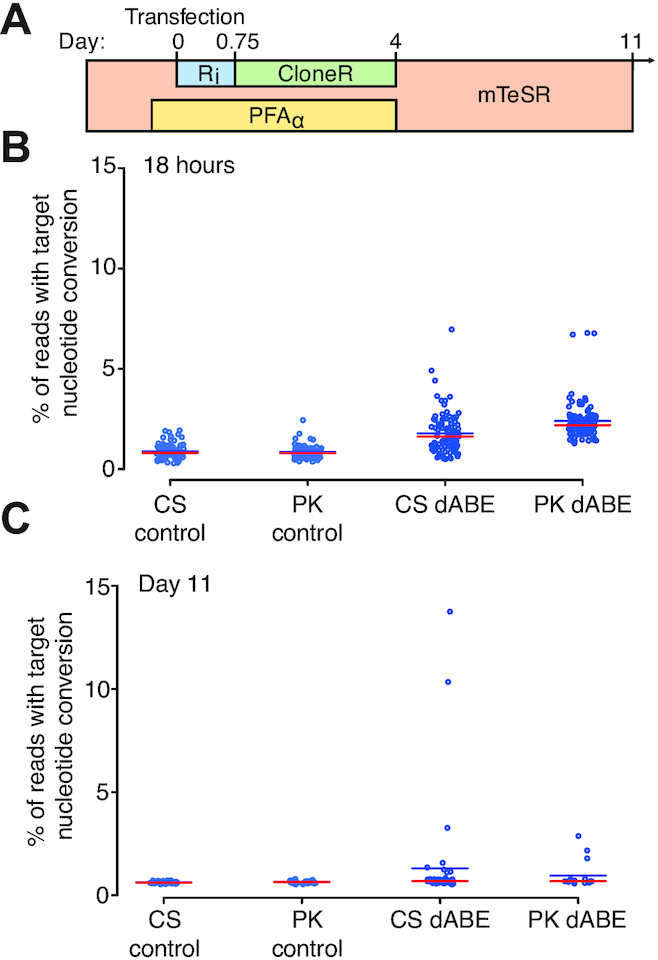
Survival cocktail and conditions for clonal derivation of iPSCs after large-scale genome engineering. (**A**) Human iPSC transfection timeline and survival cocktail conditions. (**B**) Eighteen-hour single cell direct NGS analysis of dABE targeting LINE-1. CS and PK indicate the two researchers who conducted the experiments. The red line indicates the median and the blue line the mean. (**C**) Live cell colony analysis of surviving iPSCs at day 11 post-transfection.

We next attempted the large-scale genome editing of PGP1 hiPSCs. The survival cocktail and single cell isolation timeline is shown in Figure 5A. The same experiment was conducted with two slight variations of the electroporation protocol in terms of total cells transfected and the total amount of DNA used (CS and PK conditions). Single cells were sorted and analyzed for target nucleotide conversion frequency 18 h post-electroporation and the average single cell had 2.09% target A→ G conversion while the highest edited single cell had ∼6.96% target A→G conversion or ∼1320 sites (Figure 5B). In parallel live single cells were isolated and stable cell lines formed at 11 days post-transfection. Colonies were analyzed for targeted LINE-1 A→G nucleotide conversion with a 1.30% and 0.96% mean editing frequency for CS and PK conditions, respectively, 18 h after transfection (Figure 5C). At day 11, the median editing efficiency of the CS live clones was higher than that of PK live clones in contrast to the value observed at the earlier time point, suggesting that lower initial editing efficiency may increase the viability of stably edited cell lines. The average single cell had 1.21% target A→ G conversion while the highest edited clone had a nucleotide conversion frequency of 13.75% which corresponds to 2600 sites genome wide, exceeding by three orders of magnitude the number of simultaneous edits previously recorded in iPSCs (43). The increased background that occurs in single cell direct analysis Figure 5B compared to isolation from an expanded colony Figure 5C is likely due to the necessary over-amplification required to get enough genomic material from a single cell. Similar observations were made in previous experiments using 293T cells. All other previously tested DNA editors failed to produce any detectable edits at the LINE-1 locus in human iPSCs which are sensitive to even minor DNA damage (44) and rapidly deplete cells transfected with Cas9 and TE gRNAs over time (Supplementary Figure S18).

We then integrated a set of six base editors and the HL1gR4 gRNA using the PiggyBac transposon system achieve genome editing over time to probe the current limits of genome editing while targeting LINE-1 (Supplementary Figure S19A). After doxycycline activation of the base editors a population average deamination frequency of 17.13% was observed for dABEmax and 0.78% for dCBEmax with majority of the editing occurring in the first five days of activation (Supplementary Figure S19B). All CBEs and nicking variants were barely elevated above background levels. We then sorted the dABEmax edited population from day 28 frozen stock for single cell direct DNA analysis of LINE-1 observing a 13.9% average editing frequency and a top edited cell at 64.3% or ∼12 200 sites genome wide (Supplementary Figure S19C). We performed a karyotype analysis of the original PGP1 iPSCs, after PB-dABEmax integration, and after HL1gR4 editing, all of which were normal demonstrating that large-scale genome editing can be conducted without gross chromosomal abnormalities (Supplementary Figures S20–22).

## DISCUSSION

CRISPR has recently brought about a radical transformation in the basic and applied biological research, leading to commercial applications and a multitude of clinical trials (45), and even to the controversial tests of human germline modifications (46–50). While the use of CRISPR and its myriad derivatives has greatly reduced the activation energy and technical skill required to perform genome editing several barriers limit fundamental and clinical applications: (i) The need for a custom gRNA, for each target, (ii) difficult delivery, (iii) inefficiencies once delivered, (iv) off-target errors, (v) on-target errors, (vi) the cytotoxicity of DNA damage when multiplexing beyond 62 loci (16), (vii) the limitation of insertion to sizes below 7.4kb (51), (viii) immune reactions to Cas, gRNA and vector. This study aims to develop tools that address the weakness of excessive cytotoxicity after large-scale genome editing.

Improving the actual multiplexed eukaryotic genome editing capabilities by several orders of magnitude holds the potential of revolutionizing human healthcare. Combinatorial functional genomic assays would enable the study of complex genetic traits with applications in evolutionary biology, population genetics, and human disease pathology. Multiplex editing has also permitted the development of successfully engineered cell treatments, such as the chimeric antigen receptor (CAR) therapies, which require the simultaneous editing of three target genes. Future treatments may require many more modifications to augment cancer immunotherapies, slow down oncogenic growth and reduce adverse effects, such as host-versus-graft disease. Furthermore, customizing host-versus-graft antigens in human- or nonhuman- donor tissues may require more modifications than have been made so far, for which the development of genome-wide editing technologies is needed. Special attention should be paid to the safety of the editing and its impact on the functional activity of the transplants, since donor tissues may persist in the patients for decades.

To complete genome-wide recoding and enable projects such as GP-write ultra-safe cells (30) or the de-extinction efforts to regain lost biodiversity, safe DNA editors must be developed to increase the number of genetic modifications possible by several orders of magnitude without triggering overwhelming DNA damage, as well as overcoming the delivery of multiple distinct gRNAs per cell, the latter of which we do not address is this study. C321.ΔA is a massively modified strain of *Escherichia coli* MG1655 for which all instances of the Amber stop codon were replaced (52). This has ‘freed-up’ an entire codon that can be used for synthetic biology applications such as biocontainment (53), or the inclusion of synthetic amino acids with novels functions. To attempt such a feat in the human genome will require the modification of 4438 Amber codons (33). We have shown that gene editors that do not cause double- or single-strand DNA breaks can generate a number of edits sufficient to theoretically achieve this genome recoding and pave the way toward making pan-virus resistant human cells. This could have commercial application toward cell-based production of monoclonal antibodies, recombinant protein therapeutics and synthetic meat production.

As our study demonstrates, genome-wide disruption of high copy number repetitive elements is now possible and opens new opportunities to study the ‘dark matter’ of the genome. CBEs that allow the generation of STOP codons within an open reading frame will be a great tool to probe at the functions of TEs, potentially turning observed associations with physio-pathological phenotypes into causations. For instance, large-scale inactivation of HERV-W and LINE-1 elements could help investigate their respective roles in multiple sclerosis and neurological processes. When delivering gRNAs targeting multiple independent loci, an enrichment in editing competent cells with homozygous edits at all targets was reported (54). While only using a single gRNA our distribution highly edited LINE-1 clones (Figure 4B) suggest that an editing competent cell state exists that enables up to ∼13 200 base transitions within two weeks in some small fraction of cells. Further investigation to identify and manipulate this editing competent state are warranted.

More in-depth studies will be necessary, however, to assess the impact of this massive editing on normal cell processes, since collateral damage may occur. We expect the thorough on- and off- target analysis at repetitive elements to remain a difficult task to accomplish due to their high level of polymorphism; therefore, strong biological controls as well as new experimental and bioinformatics pipelines will be needed to overcome such a challenge.

In our study, we observed that dABE increases the viability of highly edited clones as compared to dCBE. This difference may be explained by two factors. First, when using HL1gR4, CBE has three target nucleotides within its deamination window as compared to one for ABE, and as a consequence, CBE converts three times more nucleotide than ABE, potentially causing additional cytotoxicity. Second, when using CBE, the uracil N-glycosylase actively catalyzes the removal of the deaminated cytosine, generating several nicks genome-wide that promote DNA damage and potential cell death. The conversion of adenosine into inosine using ABE may not be detected as efficiently by the DNA repair machinery, therefore increasing the viability of large-scale editing. Thus, we anticipate the conditional modulation of DNA repair processes such as mismatch repair or base excision repair—that trigger downstream single- and double-strand breaks in the genome—to further improve the extent of dBEs’ performance.

At last, since dBEs do not generate direct breaks in the genome, they decrease indel frequency to background and may not trigger DNA sensors such as p53, while retaining about 34–53% targeted nucleotide conversion frequencies as compared to their nBE counterparts. 293T cells have an impaired p53 response so this isn’t likely the only explanation. We did not observe an increase in apoptotic markers in our study but an increase in the DNA damage marker $\lambda$-H2AX was observed in cells expressing Cas9 and a repetitive element targeting gRNA (55). Another possible mechanism for toxicity when nicking near high copy targets is that they could disrupt RNA expression of essential genes required for survival. As a consequence, successful genetic modifications with dBEs may not enrich for pro-oncogenic cells that have disrupted DNA-damage guardians as has been reported for Cas9 (56). Even at low levels of multiplexing, this feature may promote dBEs as an essential tool for therapeutic applications such as gene therapies.

In summary, this work optimized large-scale genome editing to enable cell viability after the simultaneous editing of thousands of loci per single cell. The ability to precisely edit many loci genome wide may facilitate the true potential of personalized medicine as we further develop our understanding of gene interactions and epistasis. We envision these new DNA editors to be combined with further improvements in the multiplex delivery of gRNAs to usher in a new phase of synthetic biology where it is possible to imagine recoding whole mammalian genomes. When combined with further modulation of DNA repair and pro-survival factors there may be no limit to the number of bases that can be modified in a single genome, opening up new avenues that previously were thought not possible. We have overcome the toxicity limitation that prevented large-scale genome editing in human iPSCs and have expanded the editing boundary by three orders of magnitude. The continued development of multiplex delivery along with non-toxic, high-efficiency DNA editors without DSBs or SSBs is paramount to the success of genome-wide recoding efforts to probe the inner workings of life, ultimately leading to the radical redesign of nature and ourselves.

## DATA AND AVAILABILITY

Key plasmids developed during this study have been submitted to Addgene: pSB700_HL1 gR4 (# 124450), dABE (# 124447) and dCBE4-gam (# 124449). All NGS data used for the figures and supplementary figures have been made available at SRA BioProject Accession #PRJNA515875, #PRJNA518077 and #PRJNA561375 for 293T, PGP1 and WGS, respectively.

## Supplementary Material

gkaa239_Supplemental_File

## References

[1] Schmid C.W., Deininger P.L. Sequence organization of the human genome. Cell. 1975; 6:345–358. 1052772 10.1016/0092-8674(75)90184-1

[2] Coufal N.G., Garcia-Perez J.L., Peng G.E., Yeo G.W., Mu Y., Lovci M.T., Morell M., O’Shea K.S., Moran J.V., Gage F.H. L1 retrotransposition in human neural progenitor cells. Nature. 2009; 460:1127–1131. 19657334 10.1038/nature08248 PMC2909034

[3] Coufal N.G., Garcia-Perez J.L., Peng G.E., Marchetto M.C.N., Muotri A.R., Mu Y., Carson C.T., Macia A., Moran J.V., Gage F.H. Ataxia telangiectasia mutated (ATM) modulates long interspersed element-1 (L1) retrotransposition in human neural stem cells. Proc. Natl. Acad. Sci. U.S.A. 2011; 108:20382–20387. 22159035 10.1073/pnas.1100273108 PMC3251057

[4] Kazazian H.H., Wong C., Youssoufian H., Scott A.F., Phillips D.G., Antonarakis S.E. Haemophilia A resulting from de novo insertion of L1 sequences represents a novel mechanism for mutation in man. Nature. 1988; 332:164–166. 2831458 10.1038/332164a0

[5] Xing J., Witherspoon D.J., Jorde L.B. Mobile element biology: new possibilities with high-throughput sequencing. Trends Genet. 2013; 29:280–289. 23312846 10.1016/j.tig.2012.12.002 PMC3938198

[6] Bodea G.O., McKelvey E.G.Z., Faulkner G.J. Retrotransposon-induced mosaicism in the neural genome. Open Biol. 2018; 8:180074. 30021882 10.1098/rsob.180074 PMC6070720

[7] Muotri A.R., Chu V.T., Marchetto M.C.N., Deng W., Moran J.V., Gage F.H. Somatic mosaicism in neuronal precursor cells mediated by L1 retrotransposition. Nature. 2005; 435:903–910. 15959507 10.1038/nature03663

[8] Muotri A.R., Marchetto M.C.N., Coufal N.G., Oefner R., Yeo G., Nakashima K., Gage F.H. L1 retrotransposition in neurons is modulated by MeCP2. Nature. 2010; 468:443–446. 21085180 10.1038/nature09544 PMC3059197

[9] De Cecco M., Criscione S.W., Peterson A.L., Neretti N., Sedivy J.M., Kreiling J.A. Transposable elements become active and mobile in the genomes of aging mammalian somatic tissues. Aging (Albany NY). 2013; 5:867–883. 24323947 10.18632/aging.100621 PMC3883704

[10] Cecco M.D., Ito T., Petrashen A.P., Elias A.E., Skvir N.J., Criscione S.W., Caligiana A., Brocculi G., Adney E.M., Boeke J.D. et al. L1 drives IFN in senescent cells and promotes age-associated inflammation. Nature. 2019; 566:73–78. 30728521 10.1038/s41586-018-0784-9 PMC6519963

[11] Wang J., Geesman G.J., Hostikka S.L., Atallah M., Blackwell B., Lee E., Cook P.J., Pasaniuc B., Shariat G., Halperin E. et al. Inhibition of activated pericentromeric SINE/Alu repeat transcription in senescent human adult stem cells reinstates self-renewal. Cell Cycle. 2011; 10:3016–3030. 21862875 10.4161/cc.10.17.17543 PMC3218602

[12] Göttle P., Förster M., Gruchot J., Kremer D., Hartung H.P., Perron H., Küry P. Rescuing the negative impact of human endogenous retrovirus envelope protein on oligodendroglial differentiation and myelination. Glia. 2018; 67:160–170. 30430656 10.1002/glia.23535

[13] Göttle P., Förster M., Gruchot J., Kremer D., Hartung H.P., Perron H., Küry P. Rescuing the negative impact of human endogenous retrovirus envelope protein on oligodendroglial differentiation and myelination. Glia. 2019; 67:160–170. 30430656 10.1002/glia.23535

[14] Kuscu C., Parlak M., Tufan T., Yang J., Szlachta K., Wei X., Mammadov R., Adli M. CRISPR-STOP: gene silencing through base-editing-induced nonsense mutations. Nat. Methods. 2017; 14:710–712. 28581493 10.1038/nmeth.4327

[15] Thompson D.B., Aboulhouda S., Hysolli E., Smith C.J., Wang S., Castanon O., Church G.M. The future of multiplexed eukaryotic genome engineering. ACS Chem. Biol. 2018; 13:313–325. 29241002 10.1021/acschembio.7b00842 PMC5880278

[16] Yang L., Güell M., Niu D., George H., Lesha E., Grishin D., Aach J., Shrock E., Xu W., Poci J. et al. Genome-wide inactivation of porcine endogenous retroviruses (PERVs). Science. 2015; 350:1101–1104. 26456528 10.1126/science.aad1191

[17] Niu D., Wei H.-J., Lin L., George H., Wang T., Lee I.-H., Zhao H.-Y., Wang Y., Kan Y., Shrock E. et al. Inactivation of porcine endogenous retrovirus in pigs using CRISPR-Cas9. Science. 2017; 357:1303–1307. 28798043 10.1126/science.aan4187 PMC5813284

[18] van Overbeek M., Capurso D., Carter M.M., Thompson M.S., Frias E., Russ C., Reece-Hoyes J.S., Nye C., Gradia S., Vidal B. et al. DNA repair profiling reveals nonrandom outcomes at Cas9-Mediated breaks. Mol. Cell. 2016; 63:633–646. 27499295 10.1016/j.molcel.2016.06.037

[19] Shen M.W., Arbab M., Hsu J.Y., Worstell D., Culbertson S.J., Krabbe O., Cassa C.A., Liu D.R., Gifford D.K., Sherwood R.I. Predictable and precise template-free CRISPR editing of pathogenic variants. Nature. 2018; 563:646–651. 30405244 10.1038/s41586-018-0686-x PMC6517069

[20] Komor A.C., Kim Y.B., Packer M.S., Zuris J.A., Liu D.R. Programmable editing of a target base in genomic DNA without double-stranded DNA cleavage. Nature. 2016; 533:420–424. 27096365 10.1038/nature17946 PMC4873371

[21] Gaudelli N.M., Komor A.C., Rees H.A., Packer M.S., Badran A.H., Bryson D.I., Liu D.R. Programmable base editing of A•T to G•C in genomic DNA without DNA cleavage. Nature. 2017; 551:464–471. 29160308 10.1038/nature24644 PMC5726555

[22] Zuo E., Sun Y., Wei W., Yuan T., Ying W., Sun H., Yuan L., Steinmetz L.M., Li Y., Yang H. Cytosine base editor generates substantial off-target single-nucleotide variants in mouse embryos. Science. 2019; 364:289–292. 30819928 10.1126/science.aav9973 PMC7301308

[23] Jin S., Zong Y., Gao Q., Zhu Z., Wang Y., Qin P., Liang C., Wang D., Qiu J.-L., Zhang F. et al. Cytosine, but not adenine, base editors induce genome-wide off-target mutations in rice. Science. 2019; 364:292–295. 30819931 10.1126/science.aaw7166

[24] Zhou C., Sun Y., Yan R., Liu Y., Zuo E., Gu C., Han L., Wei Y., Hu X., Zeng R. et al. Off-target RNA mutation induced by DNA base editing and its elimination by mutagenesis. Nature. 2019; 571:275–278. 31181567 10.1038/s41586-019-1314-0

[25] Grünewald J., Zhou R., Garcia S.P., Iyer S., Lareau C.A., Aryee M.J., Joung J.K. Transcriptome-wide off-target RNA editing induced by CRISPR-guided DNA base editors. Nature. 2019; 569:433–437. 30995674 10.1038/s41586-019-1161-z PMC6657343

[26] Komor A.C., Zhao K.T., Packer M.S., Gaudelli N.M., Waterbury A.L., Koblan L.W., Kim Y.B., Badran A.H., Liu D.R. Improved base excision repair inhibition and bacteriophage Mu Gam protein yields C:G-to-T:A base editors with higher efficiency and product purity. Sci. Adv. 2017; 3:eaao4774. 28875174 10.1126/sciadv.aao4774 PMC5576876

[27] Zhou C., Zhang M., Wei Y., Sun Y., Sun Y., Pan H., Yao N., Zhong W., Li Y., Li W. et al. Highly efficient base editing in human tripronuclear zygotes. Protein Cell. 2017; 8:772–775. 28875305 10.1007/s13238-017-0459-6 PMC5636752

[28] Chadwick A.C., Wang X., Musunuru K. In vivo base editing of PCSK9 (Proprotein Convertase Subtilisin/Kexin Type 9) as a therapeutic alternative to genome editing. Arterioscler. Thromb. Vasc. Biol. 2017; 37:1741–1747. 28751571 10.1161/ATVBAHA.117.309881 PMC5570639

[29] Li Z., Duan X., An X., Feng T., Li P., Li L., Liu J., Wu P., Pan D., Du X. et al. Efficient RNA-guided base editing for disease modeling in pigs. Cell Discov. 2018; 4:64. 30588328 10.1038/s41421-018-0065-7 PMC6297129

[30] Boeke J.D., Church G., Hessel A., Kelley N.J., Arkin A., Cai Y., Carlson R., Chakravarti A., Cornish V.W., Holt L. et al. The Genome Project-Write. Science. 2016; 353:126–127. 27256881 10.1126/science.aaf6850

[31] Milligan J.R., Aguilera J.A., Fahey R.C., Ward J.F. DNA repair by thiols in air shows two radicals make a Double-Strand break. Radiat. Res. 1995; 143:273–280. 7652164

[32] Weinfeld M., Soderlind K.J. 32P-postlabeling detection of radiation-induced DNA damage: identification and estimation of thymine glycols and phosphoglycolate termini. Biochemistry. 1991; 30:1091–1097. 1846559 10.1021/bi00218a031

[33] Sun J., Chen M., Xu J., Luo J. Relationships among stop codon usage bias, its context, isochores, and gene expression level in various eukaryotes. J. Mol. Evol. 2005; 61:437–444. 16170455 10.1007/s00239-004-0277-3

[34] Penzkofer T., Jäger M., Figlerowicz M., Badge R., Mundlos S., Robinson P.N., Zemojtel T. L1Base 2: more retrotransposition-active LINE-1s, more mammalian genomes. Nucleic Acids Res. 2017; 45:D68–D73. 27924012 10.1093/nar/gkw925 PMC5210629

[35] Grandi N., Cadeddu M., Blomberg J., Tramontano E. Contribution of type W human endogenous retroviruses to the human genome: characterization of HERV-W proviral insertions and processed pseudogenes. Retrovirology. 2016; 13:67. 27613107 10.1186/s12977-016-0301-x PMC5016936

[36] Chavez A., Scheiman J., Vora S., Pruitt B.W., Tuttle M., Iyer E.P.R., Lin S., Kiani S., Guzman C.D., Wiegand D.J. et al. Highly efficient Cas9-mediated transcriptional programming. Nat. Meth. 2015; 12:326–328. 10.1038/nmeth.3312 PMC4393883 25730490

[37] Kleinstiver B.P., Prew M.S., Tsai S.Q., Nguyen N.T., Topkar V.V., Zheng Z., Joung J.K. Broadening the targeting range of Staphylococcus aureus CRISPR-Cas9 by modifying PAM recognition. Nat. Biotechnol. 2015; 33:1293–1298. 26524662 10.1038/nbt.3404 PMC4689141

[38] Byrne S.M., Church G.M. Crispr-mediated gene targeting of human induced pluripotent stem cells. Curr. Protoc. Stem Cell Biol. 2015; 35:5A.8.1-22. 26949444 10.1002/9780470151808.sc05a08s35 PMC4772967

[39] Langmead B., Salzberg S.L. Fast gapped-read alignment with Bowtie 2. Nat. Methods. 2012; 9:357–359. 22388286 10.1038/nmeth.1923 PMC3322381

[40] Li H., Handsaker B., Wysoker A., Fennell T., Ruan J., Homer N., Marth G., Abecasis G., Durbin R. The sequence alignment/map format and SAMtools. Bioinformatics. 2009; 25:2078–2079. 19505943 10.1093/bioinformatics/btp352 PMC2723002

[41] Tarailo-Graovac M., Chen N. Using RepeatMasker to identify repetitive elements in genomic sequences. Curr. Protoc. Bioinform. 2009; 25:doi:10.1002/0471250953.bi0410s25. 10.1002/0471250953.bi0410s25 19274634

[42] Xu G.-C., Xu T.-J., Zhu R., Zhang Y., Li S.-Q., Wang H.-W., Li J.-T. LR_Gapcloser: a tiling path-based gap closer that uses long reads to complete genome assembly. Gigascience. 2019; 8:doi:10.1093/gigascience/giy157. 10.1093/gigascience/giy157 PMC6324547 30576505

[43] Riesenberg S., Chintalapati M., Macak D., Kanis P., Maricic T., Pääbo S. Simultaneous precise editing of multiple genes in human cells. Nucleic Acids Res. 2019; 47:e116. 31392986 10.1093/nar/gkz669 PMC6821318

[44] García C.P., Videla Richardson G.A., Romorini L., Miriuka S.G., Sevlever G.E., Scassa M.E. Topoisomerase I inhibitor, camptothecin, induces apoptogenic signaling in human embryonic stem cells. Stem Cell Res. 2014; 12:400–414. 24380814 10.1016/j.scr.2013.12.002

[45] Baylis F., McLeod M. First-in-human Phase 1 CRISPR gene editing cancer Trials: Are we ready. Curr. Gene Ther. 2017; 17:309–319. 29173170 10.2174/1566523217666171121165935 PMC5769084

[46] Liang P., Xu Y., Zhang X., Ding C., Huang R., Zhang Z., Lv J., Xie X., Chen Y., Li Y. et al. CRISPR/Cas9-mediated gene editing in human tripronuclear zygotes. Protein Cell. 2015; 6:363–372. 25894090 10.1007/s13238-015-0153-5 PMC4417674

[47] Kang X., He W., Huang Y., Yu Q., Chen Y., Gao X., Sun X., Fan Y. Introducing precise genetic modifications into human 3PN embryos by CRISPR/Cas-mediated genome editing. J. Assist. Reprod. Genet. 2016; 33:581–588. 27052831 10.1007/s10815-016-0710-8 PMC4870449

[48] Tang L., Zeng Y., Du H., Gong M., Peng J., Zhang B., Lei M., Zhao F., Wang W., Li X. et al. CRISPR/Cas9-mediated gene editing in human zygotes using Cas9 protein. Mol. Genet. Genomics. 2017; 292:525–533. 28251317 10.1007/s00438-017-1299-z

[49] Ma H., Marti-Gutierrez N., Park S.-W., Wu J., Lee Y., Suzuki K., Koski A., Ji D., Hayama T., Ahmed R. et al. Correction of a pathogenic gene mutation in human embryos. Nature. 2017; 548:413–419. 28783728 10.1038/nature23305

[50] Zeng Y., Li J., Li G., Huang S., Yu W., Zhang Y., Chen D., Chen J., Liu J., Huang X. Correction of the marfan syndrome pathogenic FBN1 mutation by base editing in human cells and heterozygous embryos. Mol. Ther. 2018; 26:2631–2637. 30166242 10.1016/j.ymthe.2018.08.007 PMC6224777

[51] Wang B., Li K., Wang A., Reiser M., Saunders T., Lockey R.F., Wang J.-W. Highly efficient CRISPR/HDR-mediated knock-in for mouse embryonic stem cells and zygotes. Biotechniques. 2015; 59:201–202. 26458548 10.2144/000114339

[52] Lajoie M.J., Rovner A.J., Goodman D.B., Aerni H.-R., Haimovich A.D., Kuznetsov G., Mercer J.A., Wang H.H., Carr P.A., Mosberg J.A. et al. Genomically recoded organisms expand biological functions. Science. 2013; 342:357–360. 24136966 10.1126/science.1241459 PMC4924538

[53] Mandell D.J., Lajoie M.J., Mee M.T., Takeuchi R., Kuznetsov G., Norville J.E., Gregg C.J., Stoddard B.L., Church G.M. Biocontainment of genetically modified organisms by synthetic protein design. Nature. 2015; 518:55–60. 25607366 10.1038/nature14121 PMC4422498

[54] Riesenberg S., Chintalapati M., Macak D., Kanis P., Maricic T., Pääbo S. Simultaneous precise editing of multiple genes in human cells. Nucleic Acids Res. 2019; 47:e116. 31392986 10.1093/nar/gkz669 PMC6821318

[55] Castanon O., Smith C.J., Khoshakhlagh P., Ferreira R., Güell M., Said K., Yildiz R., Dysart M., Wang S., Thompson D. et al. CRISPR-mediated biocontainment. 2020; bioRxiv doi:04 February 2020, preprint: not peer reviewed 10.1101/2020.02.03.922146.

[56] Ihry R.J., Worringer K.A., Salick M.R., Frias E., Ho D., Theriault K., Kommineni S., Chen J., Sondey M., Ye C. et al. p53 inhibits CRISPR–Cas9 engineering in human pluripotent stem cells. Nat. Med. 2018; 24:939–946. 29892062 10.1038/s41591-018-0050-6

