# Supplementary material for: Enabling large-scale genome editing at repetitive elements by reducing DNA nicking
Source: Nucleic Acids Res. 2020 Apr 21;48(9):5183–95. doi: 10.1093/nar/gkaa239 (PMC7229841; doi:10.1093/nar/gkaa239)
Supplement: gkaa239_Supplemental_File [file gkaa239_supplemental_file.pdf]

Supplementary Material for

Enabling large-scale genome editing at repetitive elements by reducing DNA nicking

Cory J. Smith[1,2,†], Oscar Castanon[1,2,3,†], Khaled Said[1,2,§], Verena Volf[1,2,§], Parastoo Khoshakhlagh[1,2,§], Amanda Hornick[1], Raphael Ferreira[4], Chun-Ting Wu[1,2], Marc Güell[5], Shilpa Garg[1], Alex Ng[1,2], Hannu Myllykallio[3], George M. Church[1,2,*].

[1] Department of Genetics, Harvard Medical School, Boston, Massachusetts, USA.

[2] Wyss Institute for Biologically Inspired Engineering, Boston, Massachusetts, USA.

[3] LOB, Ecole Polytechnique, CNRS, INSERM, Institut Polytechnique de Paris, 91128 Palaiseau, France.

[4] John A. Paulson School of Engineering and Applied Sciences, Harvard University, Cambridge, Massachusetts, USA.

[5] Department of Biology and Biological Engineering, Chalmers University of Technology, SE412 96 Gothenburg, Sweden.

[6] Pompeu Fabra University, Barcelona Biomedical Research Park, 08003-Barcelona, Spain.

[†] These authors contributed equally to this work.

[§] These authors contributed equally to this work.

[*] Corresponding author.

This PDF file includes:

Figures S1 to S22
Tables S1 to S6
Supplementary results
Data and materials availability
Supplementary references

SUPPLEMENTARY FIGURES

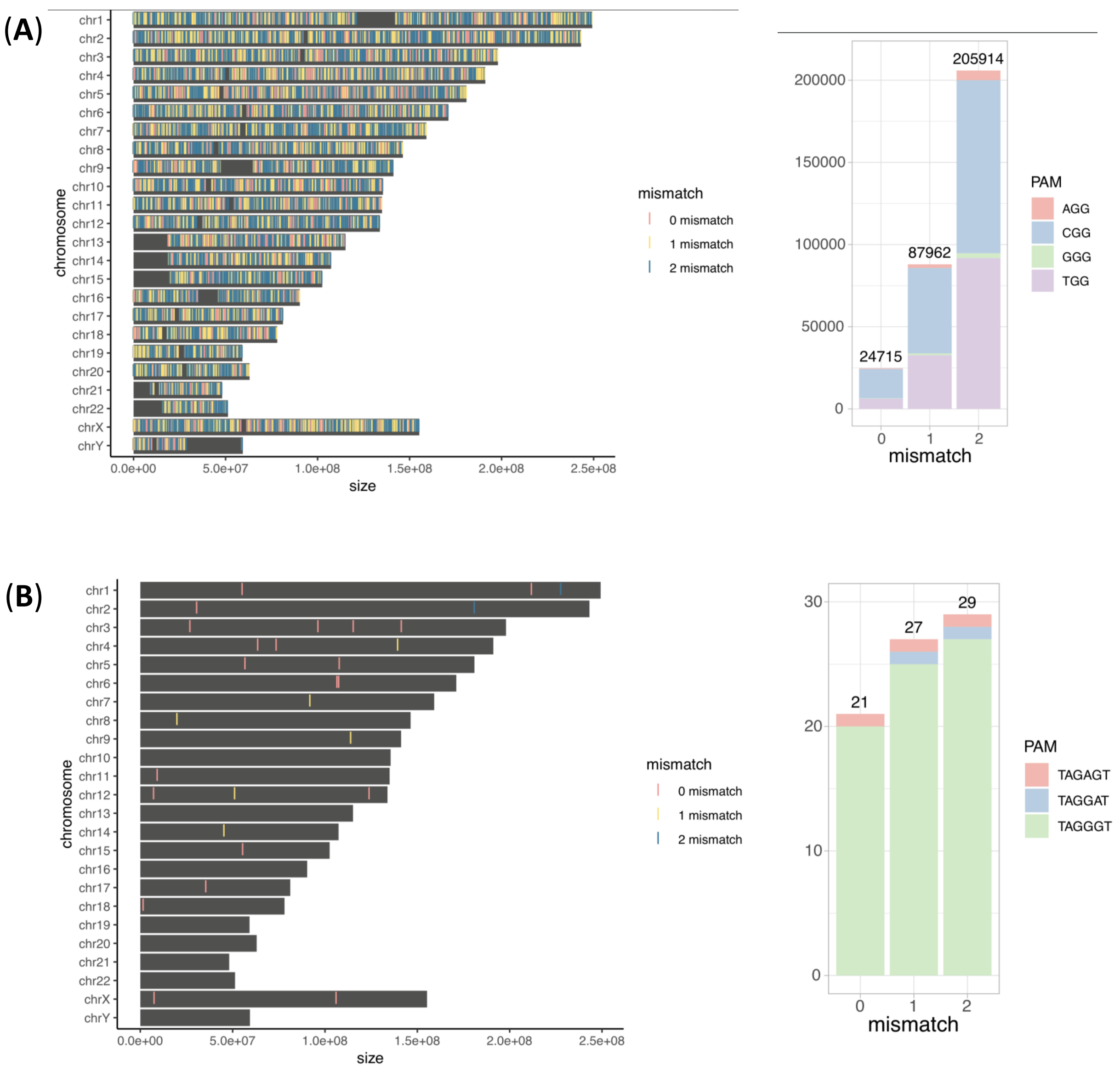

Figure S1 | TE gRNA human reference alignment. (**A**) (left) Genome wide distribution of gRNA Alu (right) Alu copy number and PAM distribution. (**B**) (left) Genome wide distribution of gRNA HERV env11 (right) HERV copy number and PAM distribution.

A)

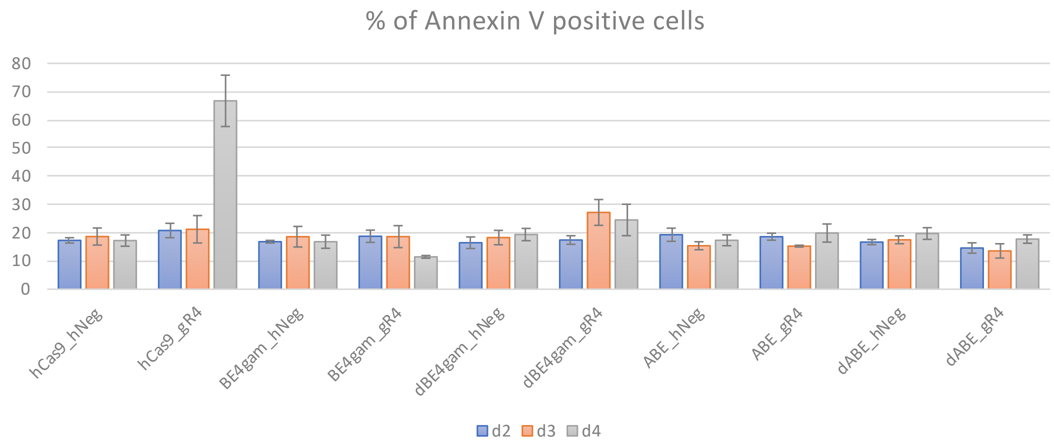

B)

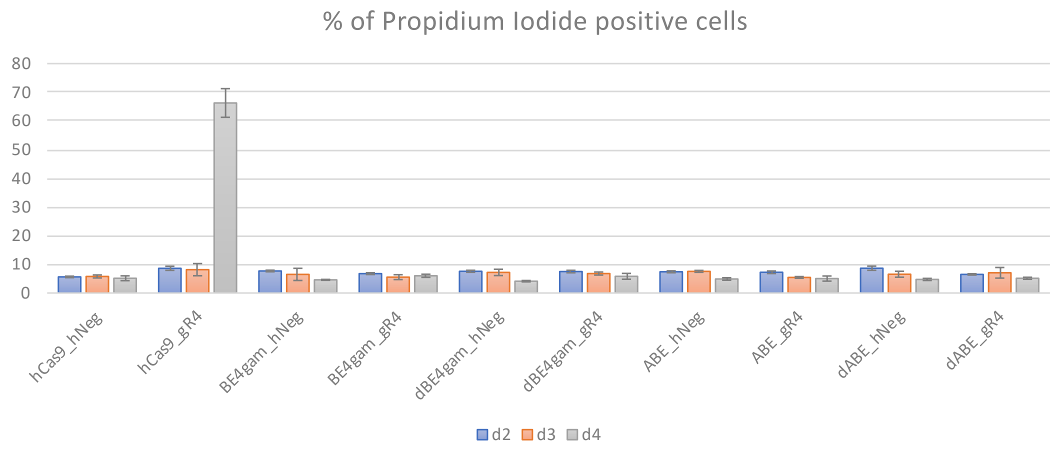

C)

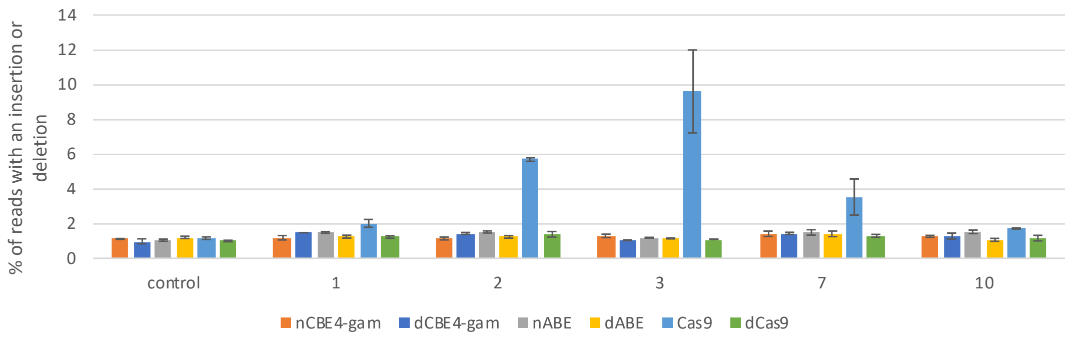

Figure S2 | Annexin V and propidium iodide assays for cytotoxicity. (A) Apoptosis cell death analysis using Annexin V targeting LINE-1. **(B)** Necrosis cell death analysis using propidium iodide. (**C**) Indel mutagenesis analysis with experimental day on the x-axis.

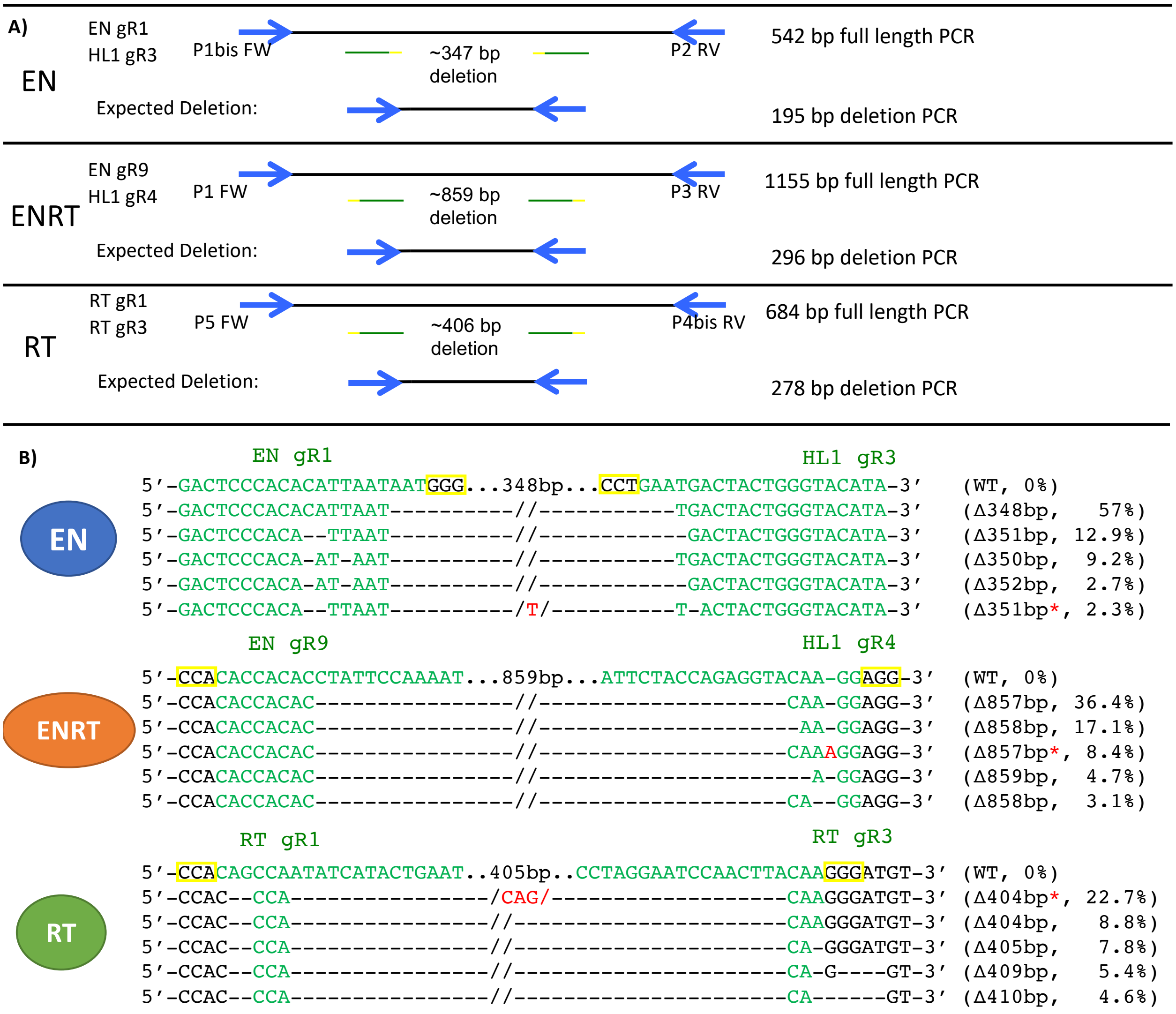

Figure S3 | dual gRNA LINE-1 deletions. (**A**) Primers used to amplify dual gRNA pairs targeting LINE-1 with full length and expected deletion product sizes shown. gRNAs are represented as green with yellow PAMs, primers are in blue. (**B**) Dual gRNA deletion frequency displaying the expected cut points near each gRNA. Green nucleotides are within the gRNA sequence, red are inserted nucleotides and "-" are deletions. The sizes of deletions and percentage among sequencing reads are displayed to the right.

A)

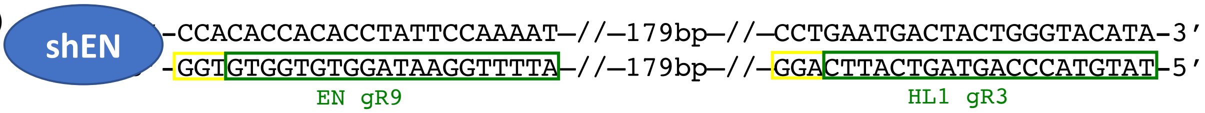

B)

condition	total cells screened	# of edited cells	% of edited cells
All samples	364	303	83.24

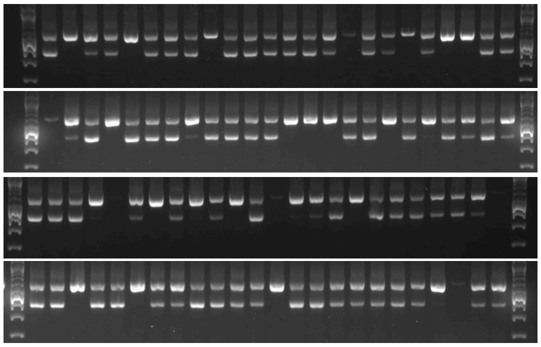

C)

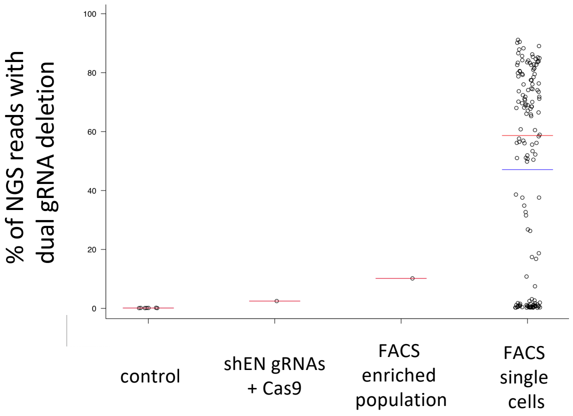

Figure S4 | Single cell analysis of dual gRNA deletions targeting LINE-1 (A) gRNA targets used for the shEN dual deletion. (**B**) Gel visualization of dual gRNA deletions bands in FACS single cells with a summary table. (**C**) Percentage of single cells with dual gRNA deletions.

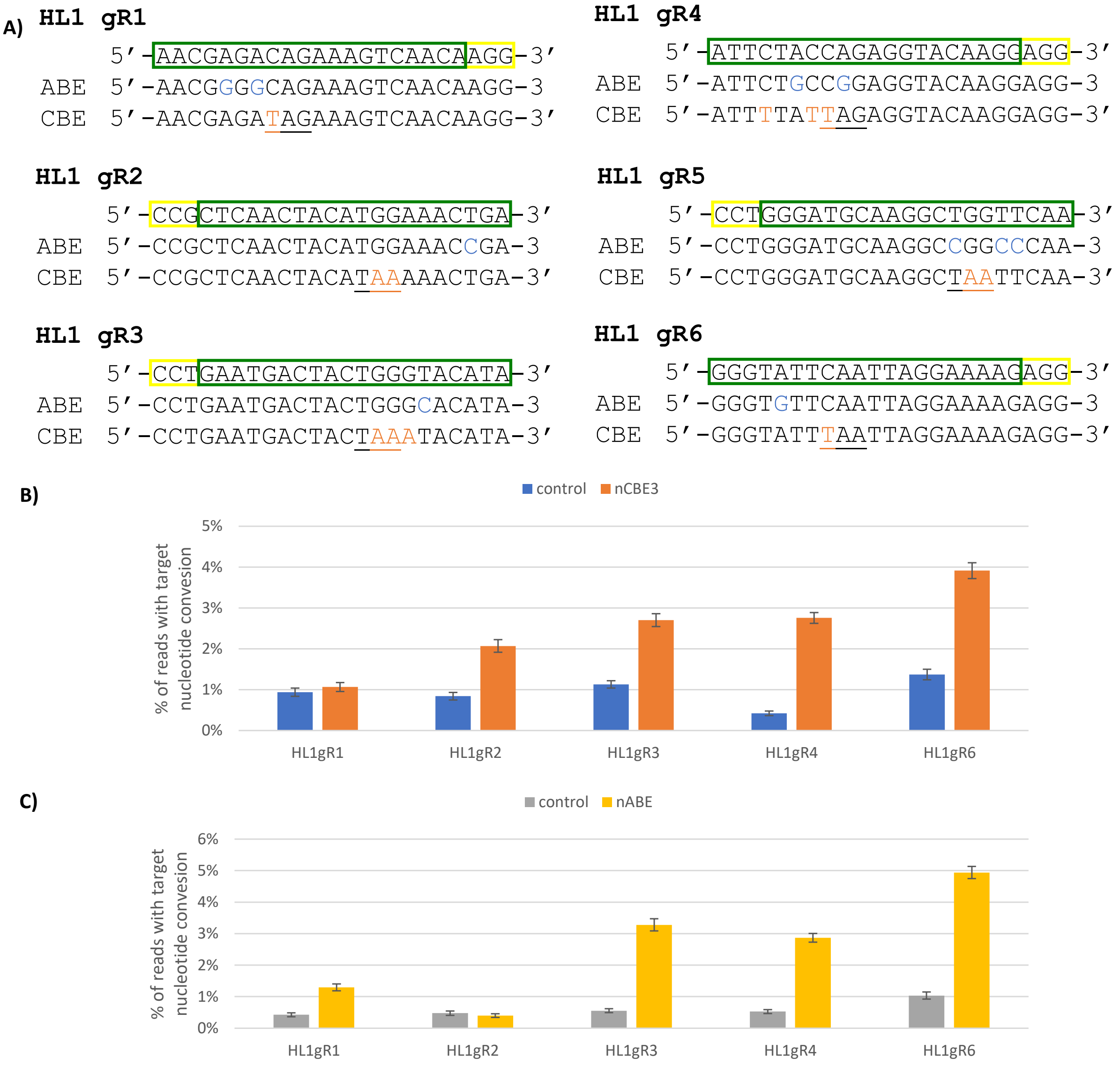

Figure S5 | nBE targeting LINE-1 (A) LINE-1 gRNA targets outlined in green boxes with PAMs in yellow. Expected ABE and CBE deamination products are displayed below with altered bases in blue and orange respectively **(B)** Targeted deamination frequency at C8 using nCBE3. **(C)** Targeted deamination frequency at A6 using nABE.

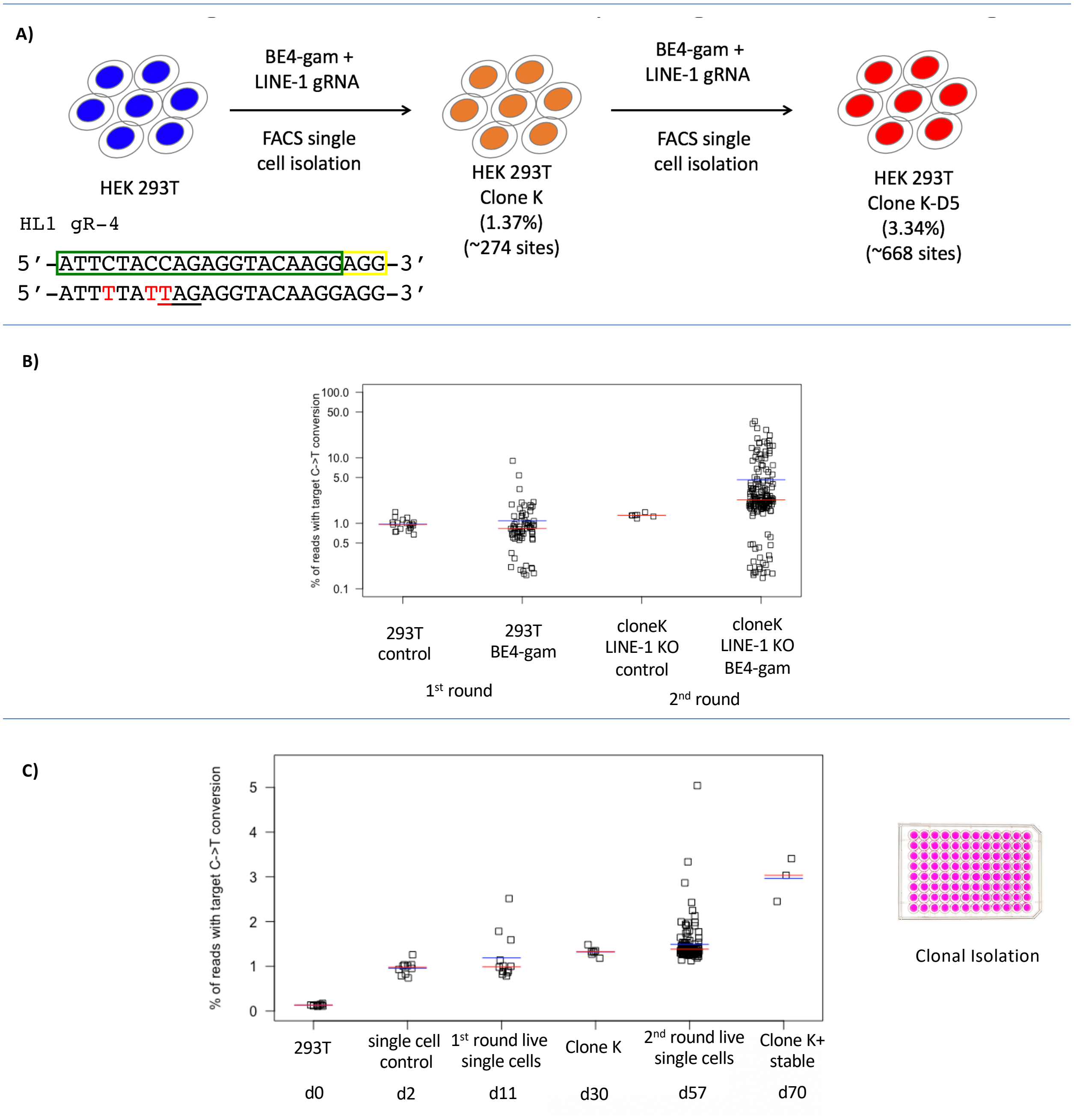

Figure S6 | Utilizing high copy repetitive elements for the testing of DNA editors. (A) Experimental design for two rounds of base editing at LINE-1. gRNA target is outlined in green with a yellow PAM. C->T deamination targets are colored in red. **(C)** Targeted deamination frequency at C8 using nCBE4-gam, direct cell analysis. **(C)** Targeted deamination frequency at C8 using nCBE4-gam over two rounds of transfection and clonal isolation.

A)

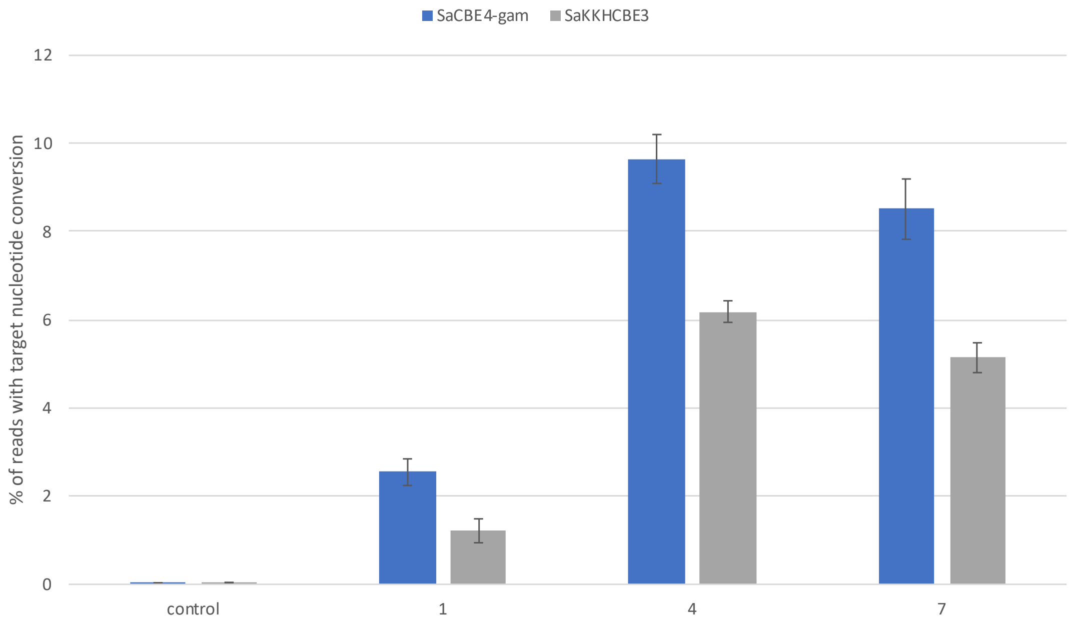

Figure S7 | Base editing at HERV. (A) Targeted deamination frequencies at C12 using a set of SaCas9-BEs over three time points (day 1, 4 and 7). Error bars represent SEM, n=3.

A)

wt

	T_3	C_4	T_5	A_6	C_7	C_8	A_9
G	0.00	0.00	0.00	0.80	0.40	0.40	0.40
A	0.00	0.80	0.00	98.80	0.40	0.40	99.20
T	99.20	1.60	98.80	0.00	2.80	0.40	0.00
C	0.80	97.60	0.80	0.40	96.40	98.90	0.40

cK

	T_3	C_4	T_5	A_6	C_7	C_8	A_9
G	0.3	0.3	0.0	1.5	0.3	0.6	0.7
A	0.0	0.0	0.0	97.4	0.7	0.7	99.3
T	99.4	2.1	97.8	0.3	5.1	2.7	0.0
C	0.3	97.6	2.2	0.8	94.0	96.0	0.0

cKA5

	T_3	C_4	T_5	A_6	C_7	C_8	A_9
G	0.6	0.3	0.0	2.2	1.0	1.3	1.1
A	0.0	0.0	0.0	97.1	1.0	1.0	98.9
T	98.7	1.3	98.6	0.6	4.1	1.9	0.0
C	0.6	98.4	1.4	0.0	93.8	95.7	0.0

cKD5

	T_3	C_4	T_5	A_6	C_7	C_8	A_9
G	0.7	0.0	0.0	0.3	0.7	0.4	0.4
A	0.4	1.1	0.0	99.3	0.4	0.7	99.2
T	98.6	3.6	99.6	0.0	4.7	3.7	0.0
C	0.3	95.3	0.4	0.3	93.9	95.2	0.4

B)

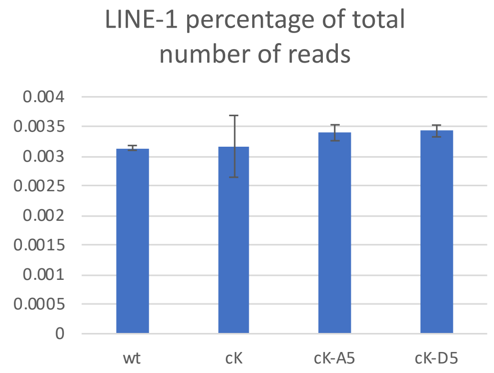

C)

	clone K vs. wt	cKA5 vs. wt	cKD5 vs. wt
downregulated genes	3055	2721	3012
not significant	7442	8114	7555
upregulated genes	2913	2591	2848

D)

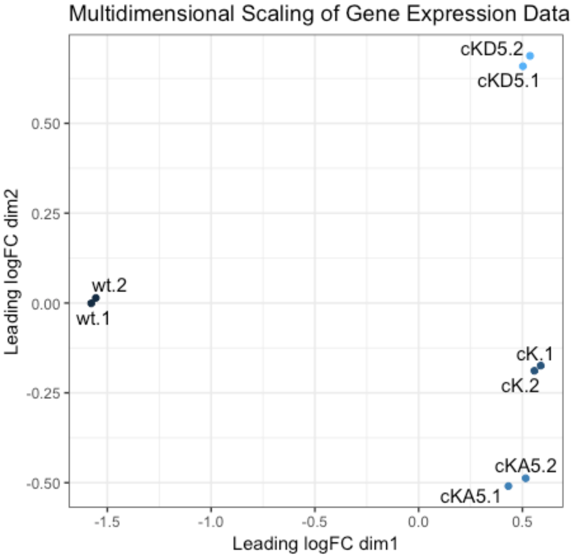

Figure S8 | LINE-1 RNA expression in KO clones (A) Base editing activity detected in RNA transcripts of clone K (cK), clone K-A5 (cKA) and clone K-D4 (cKD5) within the gRNA target sequence **(B)** Percentage of LINE-1 reads relative to total number of reads. Error bars represent standard deviation between biological duplicates. **(C)** Summary of differentially expressed genes as determined by the exact test. **(D)** Multidimensional scaling plot where distance corresponds to leading log-fold count changes between the RNA samples.

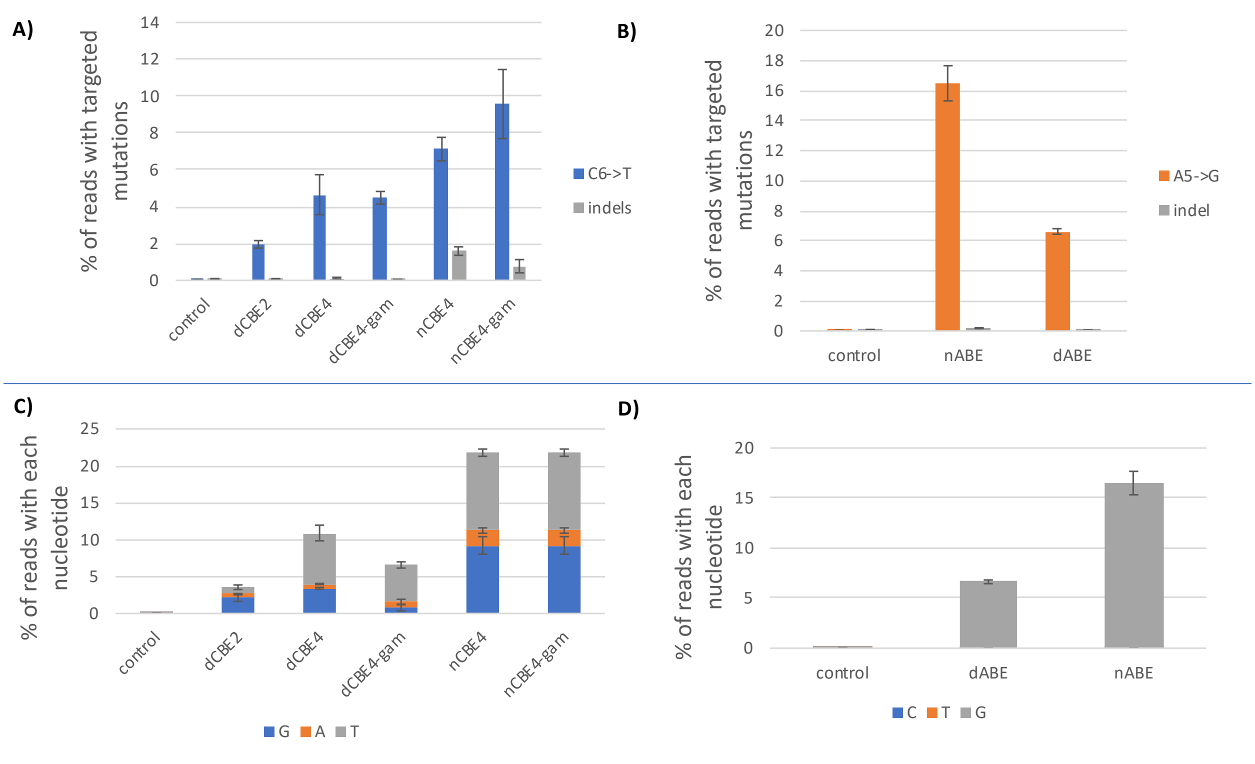

Figure S9 | dBE vs nBE at a single locus target. (A) Targeted deamination and indel frequencies at C6 using a set of CBEs and gRNA S1. Error bars represent SEM, n=3. **(B)** Targeted deamination and indel frequencies at A5 using ABEs.(**C**) Base editing purity analysis of C6. (**D**) Purity analysis for A5 using ABEs.

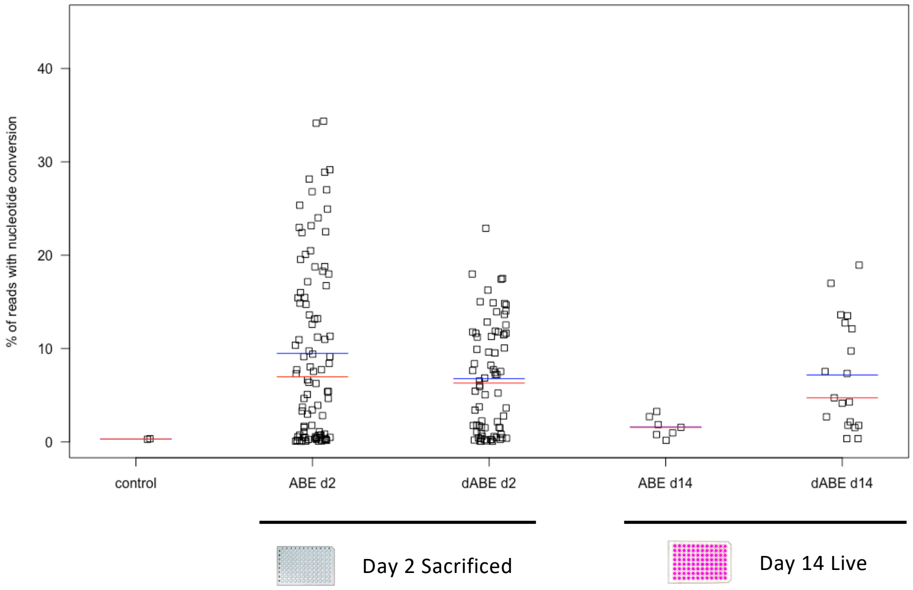

Figure S10 | dABE targeting LINE-1 single cell analysis. **(A)** Base editing in HEK 293Ts after transfection comparing nABE vs dABE with HL1gR46 at days 2 and 14. FACS single cells are plotted as individual points representing targeted base editing nucleotide deamination. Red line indicates the median and the blue line the mean.

A)

control

																						PAM	
	A_1	T_2	T_3	C_4	T_5	A_6	C_7	C_8	A_9	G_{10}	A_{11}	G_{12}	G_{13}	T_{14}	A_{15}	C_{16}	A_{17}	A_{18}	G_{19}	G_{20}	A	G	G
G	0.3	0.1	0.2	0.2	0.1	0.6	0.3	0.4	0.3	98.1	0.3	98.3	97.7	0.1	1.0	1.0	0.7	0.6	96.9	97.7	0.4	97.7	98.6
A	99.2	0.2	0.2	0.2	0.1	98.6	0.9	0.4	99.2	0.5	99.1	0.9	1.2	0.1	98.6	1.2	98.8	97.8	2.8	1.4	99.4	1.8	0.7
T	0.3	99.7	99.3	0.6	99.0	0.2	3.5	0.4	0.3	0.2	0.3	0.4	0.7	99.1	0.2	1.9	0.2	1.5	0.3	0.4	0.1	0.4	0.4
C	0.1	0.3	0.3	98.9	0.7	0.4	95.1	98.7	0.1	1.0	0.2	0.6	0.4	0.6	0.1	96.1	0.3	0.2	0.1	0.5	0.1	0.2	0.3

nCBE4-gam

																						PAM	
	A_1	T_2	T_3	C_4	T_5	A_6	C_7	C_8	A_9	G_{10}	A_{11}	G_{12}	G_{13}	T_{14}	A_{15}	C_{16}	A_{17}	A_{18}	G_{19}	G_{20}	A	G	G
G	0.3	0.0	0.1	0.5	0.1	0.6	0.4	0.5	0.3	98.1	0.3	98.4	97.9	0.1	1.1	0.7	0.6	0.5	96.8	98.2	0.3	97.9	98.2
A	99.3	0.1	0.1	0.2	0.1	98.7	0.9	0.4	99.2	0.6	99.3	0.8	1.2	0.1	98.5	1.2	99.0	97.9	2.9	1.1	99.4	1.7	0.9
T	0.2	99.7	99.5	7.4	99.3	0.2	9.9	6.9	0.3	0.1	0.3	0.3	0.6	99.2	0.3	2.1	0.1	1.4	0.2	0.2	0.2	0.3	0.5
C	0.2	0.2	0.2	91.8	0.4	0.4	88.7	92.2	0.1	1.1	0.1	0.3	0.2	0.6	0.1	96.0	0.2	0.2	0.1	0.5	0.1	0.2	0.4

dCBE4-gam

																						PAM	
	A_1	T_2	T_3	C_4	T_5	A_6	C_7	C_8	A_9	G_{10}	A_{11}	G_{12}	G_{13}	T_{14}	A_{15}	C_{16}	A_{17}	A_{18}	G_{19}	G_{20}	A	G	G
G	0.3	0.0	0.0	1.1	0.0	0.3	0.6	0.2	0.2	97.7	0.1	98.6	98.3	0.0	0.7	1.4	0.6	0.6	96.3	97.8	0.4	97.6	98.6
A	99.5	0.0	0.1	0.2	0.1	98.5	1.3	0.4	99.1	0.7	99.4	0.6	1.1	0.1	98.7	1.3	99.3	97.3	3.3	1.7	99.4	1.9	0.9
T	0.1	99.7	99.5	23.9	99.0	0.2	25.8	24.2	0.3	0.3	0.1	0.3	0.4	99.2	0.3	2.2	0.2	1.8	0.3	0.1	0.2	0.5	0.2
C	0.0	0.2	0.1	74.3	0.5	0.4	71.8	74.9	0.0	0.9	0.1	0.3	0.2	0.4	0.1	96.4	0.1	0.3	0.0	0.3	0.0	0.1	0.3

nABE

																						PAM	
	A_1	T_2	T_3	C_4	T_5	A_6	C_7	C_8	A_9	G_{10}	A_{11}	G_{12}	G_{13}	T_{14}	A_{15}	C_{16}	A_{17}	A_{18}	G_{19}	G_{20}	A	G	G
G	0.3	0.1	0.1	0.2	0.3	1.3	0.3	0.4	0.2	98.4	0.3	98.4	97.6	0.1	1.0	0.9	0.8	0.6	96.4	97.9	0.4	97.4	98.5
A	99.2	0.1	0.2	0.2	0.1	97.9	0.8	0.5	99.3	0.4	99.3	0.9	1.3	0.1	98.6	1.4	98.7	97.4	3.2	1.4	99.2	1.9	0.9
T	0.3	99.7	99.4	0.5	98.8	0.2	3.6	0.4	0.3	0.2	0.2	0.6	0.6	99.0	0.2	1.9	0.3	1.7	0.3	0.2	0.2	0.5	0.3
C	0.1	0.2	0.2	99.0	0.8	0.5	95.1	98.6	0.1	0.9	0.1	0.5	0.4	0.7	0.1	96.2	0.3	0.3	0.0	0.5	0.0	0.2	0.3

dABE

																						PAM	
	A_1	T_2	T_3	C_4	T_5	A_6	C_7	C_8	A_9	G_{10}	A_{11}	G_{12}	G_{13}	T_{14}	A_{15}	C_{16}	A_{17}	A_{18}	G_{19}	G_{20}	A	G	G
G	0.4	0.1	0.1	0.2	0.1	49.6	0.4	0.4	2.3	98.4	0.3	98.6	97.9	0.1	0.8	0.7	0.7	0.5	96.9	97.9	0.3	97.8	98.7
A	99.3	0.1	0.2	0.1	0.1	49.7	0.7	0.4	97.3	0.5	99.2	0.6	1.2	0.0	98.7	1.1	98.9	97.9	2.8	1.2	99.5	1.7	0.7
T	0.2	99.7	99.4	0.5	99.2	0.2	3.3	0.4	0.3	0.1	0.3	0.6	0.6	99.3	0.4	1.9	0.1	1.5	0.3	0.4	0.2	0.3	0.3
C	0.1	0.2	0.3	99.0	0.6	0.5	95.5	98.8	0.1	0.9	0.2	0.5	0.4	0.5	0.1	96.4	0.2	0.1	0.0	0.5	0.0	0.2	0.3

Figure S11 | (A) Base editing window comparing ABE vs CBE and nCas9-BE vs dCas9-BE in the top edited live single cell isolated stable cell line.

A)

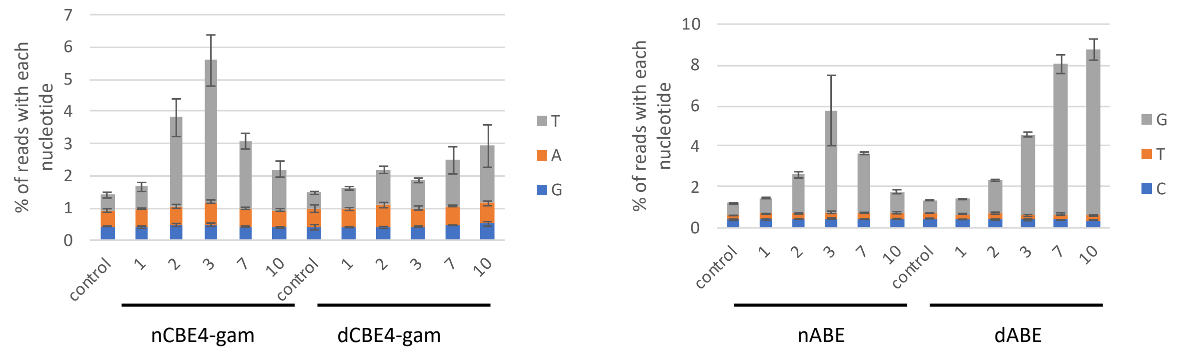

B)

HL1gR4 population control

																					PAM		Indel %
	A_1	T_2	T_3	C_4	T_5	A_6	C_7	C_8	A_9	G_{10}	A_{11}	G_{12}	G_{13}	T_{14}	A_{15}	C_{16}	A_{17}	A_{18}	G_{19}	G_{20}	A	G	G	1.22
G	0.2	0.1	0.1	0.3	0.1	0.6	0.4	0.3	0.3	98.3	0.2	98.2	97.8	0.0	1.1	1.0	0.8	0.4	95.9	97.7	0.4	97.6	98.7	
A	99.5	0.1	0.3	0.1	0.1	98.7	0.9	0.7	99.3	0.5	99.2	1.1	1.2	0.0	98.7	1.4	98.8	98.1	3.8	1.6	99.4	1.9	0.9	
T	0.2	99.7	99.4	0.4	99.3	0.2	3.6	0.5	0.3	0.2	0.3	0.3	0.6	99.2	0.1	1.8	0.2	1.2	0.2	0.3	0.1	0.5	0.3	
C	0.0	0.2	0.2	99.2	0.5	0.4	95.0	98.5	0.1	1.0	0.2	0.5	0.5	0.7	0.1	95.9	0.2	0.3	0.1	0.4	0.1	0.2	0.2	

nCBE4-gam

																					PAM		Indel %
	A_1	T_2	T_3	C_4	T_5	A_6	C_7	C_8	A_9	G_{10}	A_{11}	G_{12}	G_{13}	T_{14}	A_{15}	C_{16}	A_{17}	A_{18}	G_{19}	G_{20}	A	G	G	1.41
G	0.2	0.0	0.2	0.4	0.1	0.5	0.4	0.4	0.4	98.1	0.3	98.4	97.6	0.2	0.9	1.0	0.7	0.5	95.1	98.0	0.4	97.5	98.4	
A	99.4	0.2	0.2	0.4	0.1	98.9	0.9	0.6	99.2	0.6	99.1	0.9	1.3	0.1	98.8	1.2	98.8	98.1	4.4	1.4	99.5	2.1	1.0	
T	0.3	99.6	99.3	2.9	99.1	0.1	5.5	2.5	0.3	0.2	0.3	0.3	0.6	99.2	0.2	1.6	0.1	1.1	0.3	0.3	0.1	0.3	0.2	
C	0.1	0.2	0.3	96.2	0.7	0.4	93.0	96.5	0.1	1.0	0.2	0.3	0.5	0.5	0.1	96.3	0.3	0.3	0.1	0.4	0.1	0.2	0.4	

dCBE4-gam

																					PAM		Indel %
	A_1	T_2	T_3	C_4	T_5	A_6	C_7	C_8	A_9	G_{10}	A_{11}	G_{12}	G_{13}	T_{14}	A_{15}	C_{16}	A_{17}	A_{18}	G_{19}	G_{20}	A	G	G	1.43
G	0.3	0.1	0.2	0.4	0.1	0.7	0.5	0.4	0.3	97.8	0.3	96.8	96.7	0.2	1.1	1.0	0.9	0.5	91.5	97.0	0.5	95.9	98.0	
A	99.2	0.1	0.3	0.3	0.2	98.6	0.9	0.6	99.3	0.8	99.1	1.2	1.8	0.1	98.4	1.5	98.6	98.0	8.2	1.8	99.3	3.5	1.4	
T	0.3	99.5	99.2	1.7	99.0	0.2	4.6	1.5	0.3	0.2	0.4	1.4	1.0	98.9	0.4	2.3	0.2	1.2	0.3	0.4	0.2	0.4	0.3	
C	0.1	0.4	0.3	97.6	0.7	0.5	93.9	97.4	0.1	1.1	0.1	0.6	0.5	0.8	0.1	95.2	0.3	0.2	0.1	0.9	0.1	0.2	0.3	

nABE

																					PAM		Indel %
	A_1	T_2	T_3	C_4	T_5	A_6	C_7	C_8	A_9	G_{10}	A_{11}	G_{12}	G_{13}	T_{14}	A_{15}	C_{16}	A_{17}	A_{18}	G_{19}	G_{20}	A	G	G	1.49
G	0.5	0.1	0.2	0.5	0.1	3.0	0.4	0.4	0.5	97.9	0.3	97.3	96.8	0.1	1.0	1.2	1.0	0.6	90.1	96.8	0.6	96.7	97.9	
A	99.1	0.3	0.2	0.4	0.2	96.2	0.9	0.6	98.9	0.9	98.9	1.4	2.0	0.2	98.5	1.5	98.6	98.0	9.5	2.1	99.1	2.7	1.5	
T	0.3	99.4	99.3	0.7	98.7	0.3	3.4	0.7	0.4	0.2	0.4	0.7	0.8	98.5	0.4	2.2	0.3	1.1	0.4	0.4	0.2	0.4	0.3	
C	0.1	0.4	0.3	98.3	0.9	0.5	95.1	98.3	0.1	1.1	0.2	0.5	0.5	1.2	0.1	95.3	0.2	0.3	0.1	0.7	0.1	0.2	0.4	

dABE

																					PAM		Indel %
	A_1	T_2	T_3	C_4	T_5	A_6	C_7	C_8	A_9	G_{10}	A_{11}	G_{12}	G_{13}	T_{14}	A_{15}	C_{16}	A_{17}	A_{18}	G_{19}	G_{20}	A	G	G	1.41
G	0.4	0.1	0.2	0.4	0.1	8.2	0.5	0.5	0.9	97.7	0.3	96.8	96.3	0.1	1.0	1.2	0.9	0.5	89.0	96.8	0.4	96.2	97.7	
A	99.2	0.3	0.3	0.3	0.2	91.1	0.9	0.5	98.6	0.8	99.0	1.5	2.0	0.1	98.5	1.6	98.7	98.2	10.7	1.8	99.3	3.2	1.5	
T	0.3	99.4	99.1	0.8	98.8	0.3	3.4	0.7	0.3	0.2	0.4	1.1	1.0	98.8	0.4	2.3	0.3	1.0	0.4	0.3	0.2	0.4	0.3	
C	0.1	0.4	0.3	98.4	0.9	0.4	95.1	98.3	0.1	1.2	0.2	0.6	0.6	1.0	0.1	95.0	0.2	0.3	0.1	1.0	0.1	0.2	0.4	

Figure S12 | Base editing purity in HEK 293T targeting LINE-1. (A) Purity of deamination at target nucleotide (left) CBE, (right) ABE. X-axis represents the day analyzed **(B)** Base editing activity across gRNA target sequence at day seven.

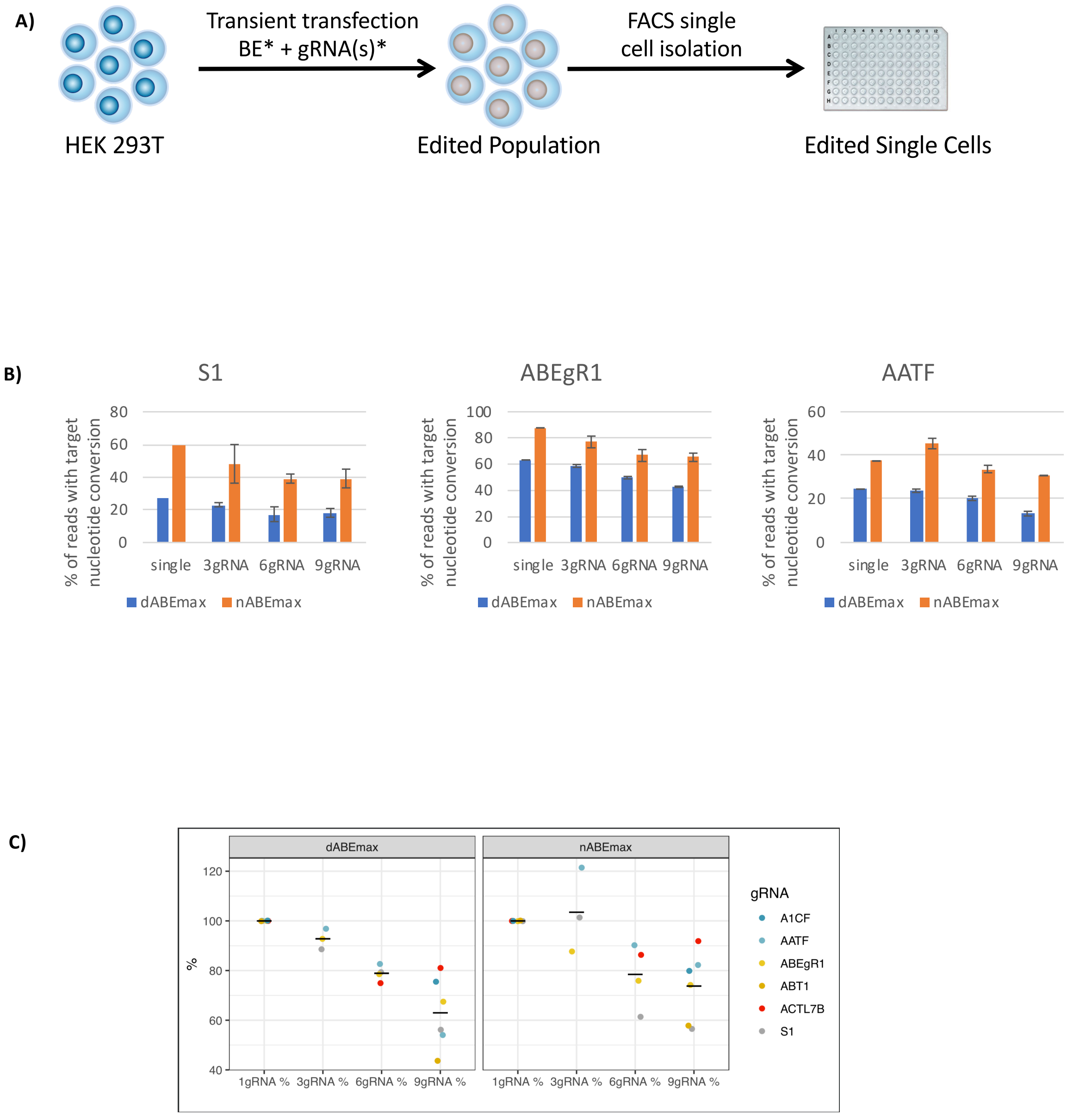

Figure S13| Multiple single locus targeting gRNA delivery with BEs (A) Diagram of the experimental workflow to deliver guides in pools of 1, 3, 6 or 9 individual gRNAs with either dABEmax or nABEmax. **(B)** Efficiency of genome editing comparing single delivery to multiple gRNA delivery plotting a single target site for each plot comparing single delivery versus multiplex. Error bars represent SEM, n=2. **(C)** Normalized editing efficiency compared to single gRNA delivery within each individual gRNA.

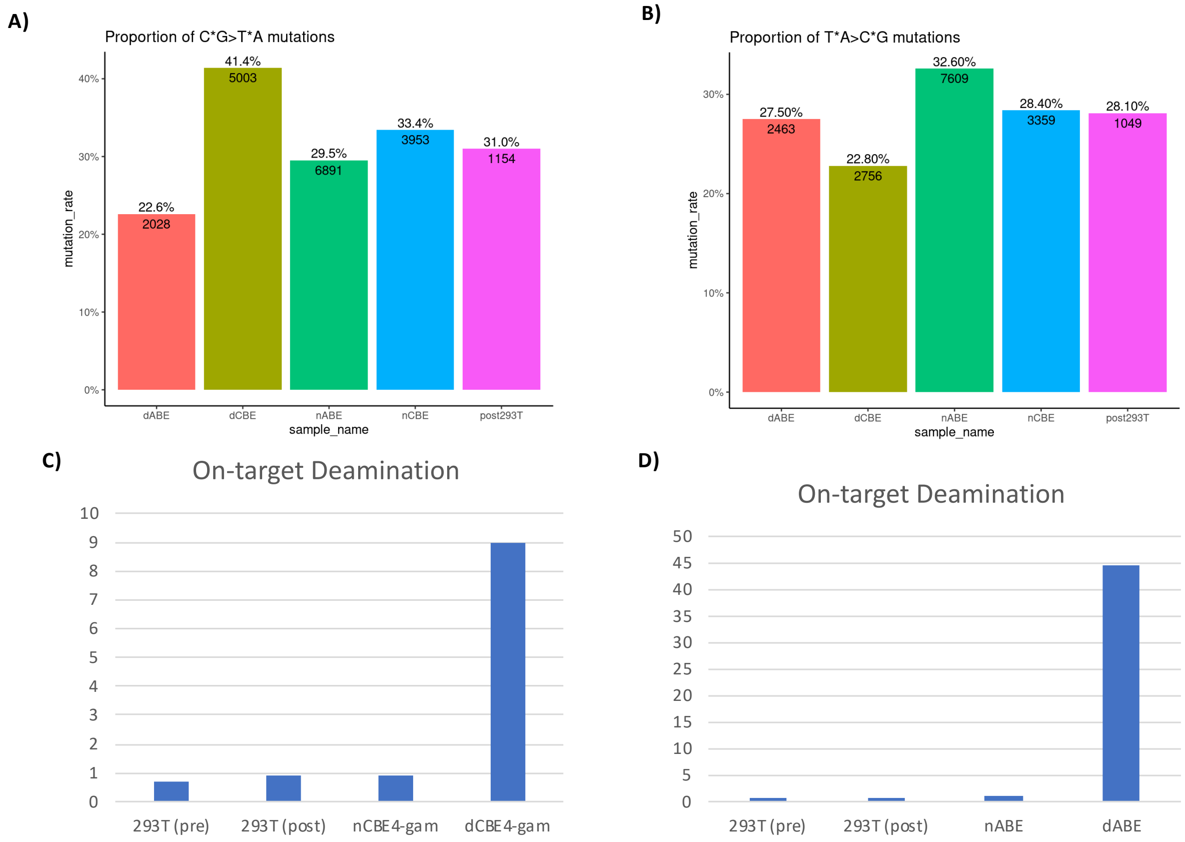

Figure S14| Genome wide off-target and on-target analysis (A) Whole Genome Sequencing analysis of the top edited 293T HL1gR4 edited clones from each of the four BEs tested. This displays the mutation spectrum observed for C*G>T*A mutations for each sample when compared to pre293T (baseline control). Each represents a single clone **(B)** This displays the mutation spectrum observed for T*A>C*G mutations each sample when compared to pre293T. **(C)** On-target LINE-1 deamination for CBE clones and controls. **(D)** On-target LINE-1 deamination for ABE clones and controls.

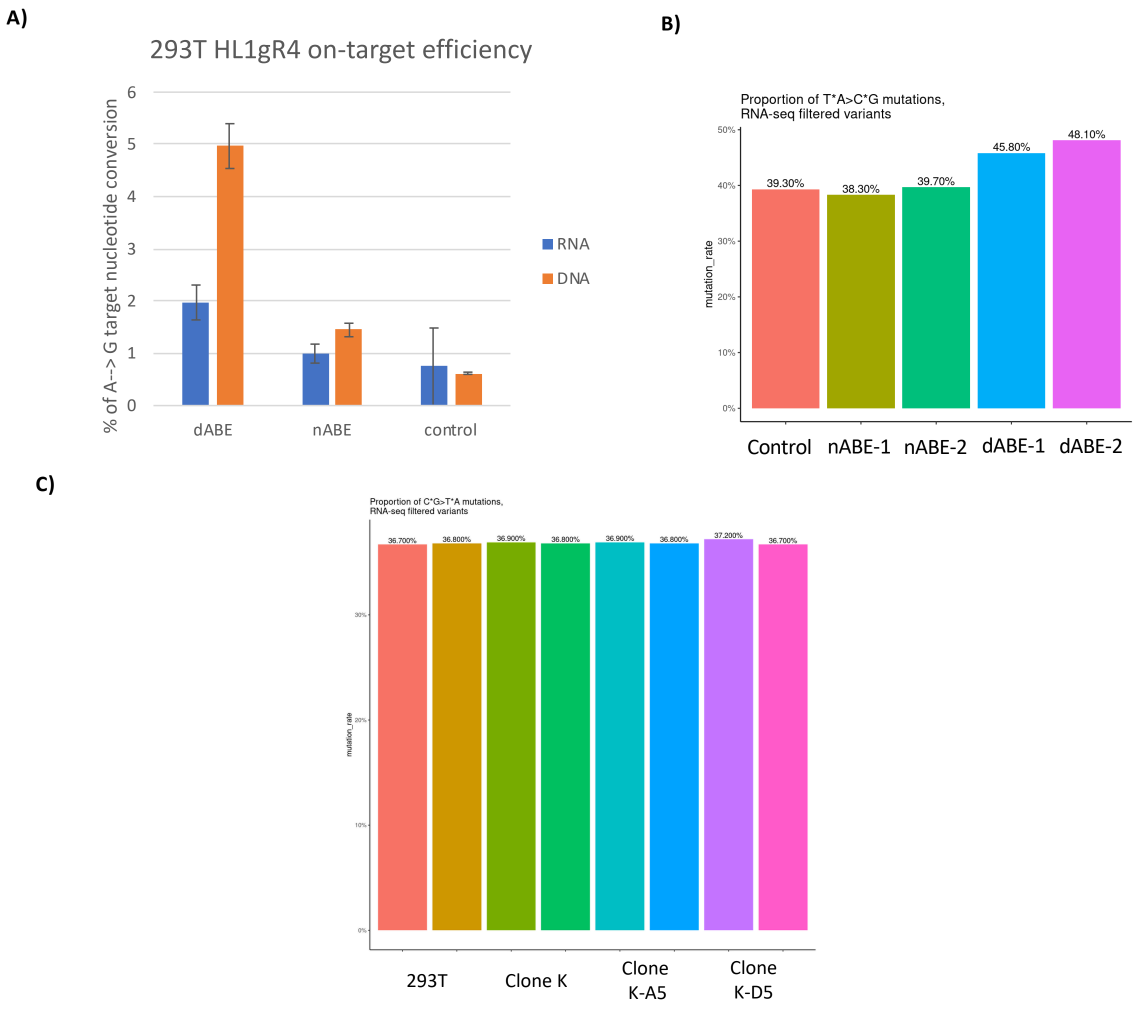

Figure S15| Genome wide off-target and on-target RNA analysis (A) RNA sequencing analysis compared to targeted LINE-1 amplicon sequencing for 293T cell transfected with BE and gRNA after two days. **(B)** RNA-seq off target analysis displays the mutation spectrum observed for T*A>C*G mutations each sample. **(C)** The C*G>T*A mutation spectrum of CBE edited clones after 30 – 70 days.

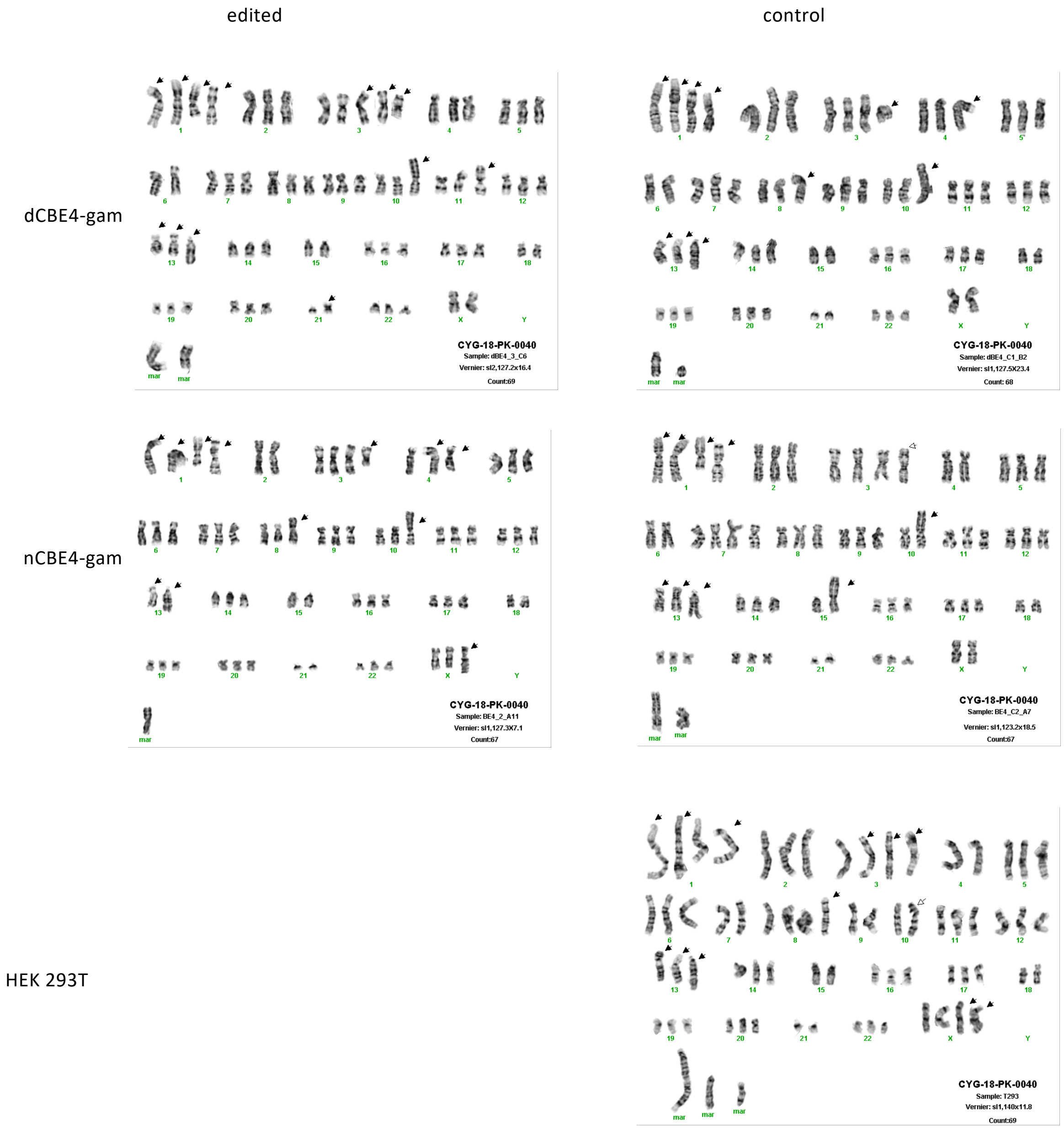

Figure S16 | Karyotype analysis after CBE4-gam editing. (A) Karyotype chromosome presentations for each of the genome edited clones and controls. The white arrows (hollow arrows) indicate non-clonal abnormalities and black arrows (solid arrows) indicate clonal abnormalities

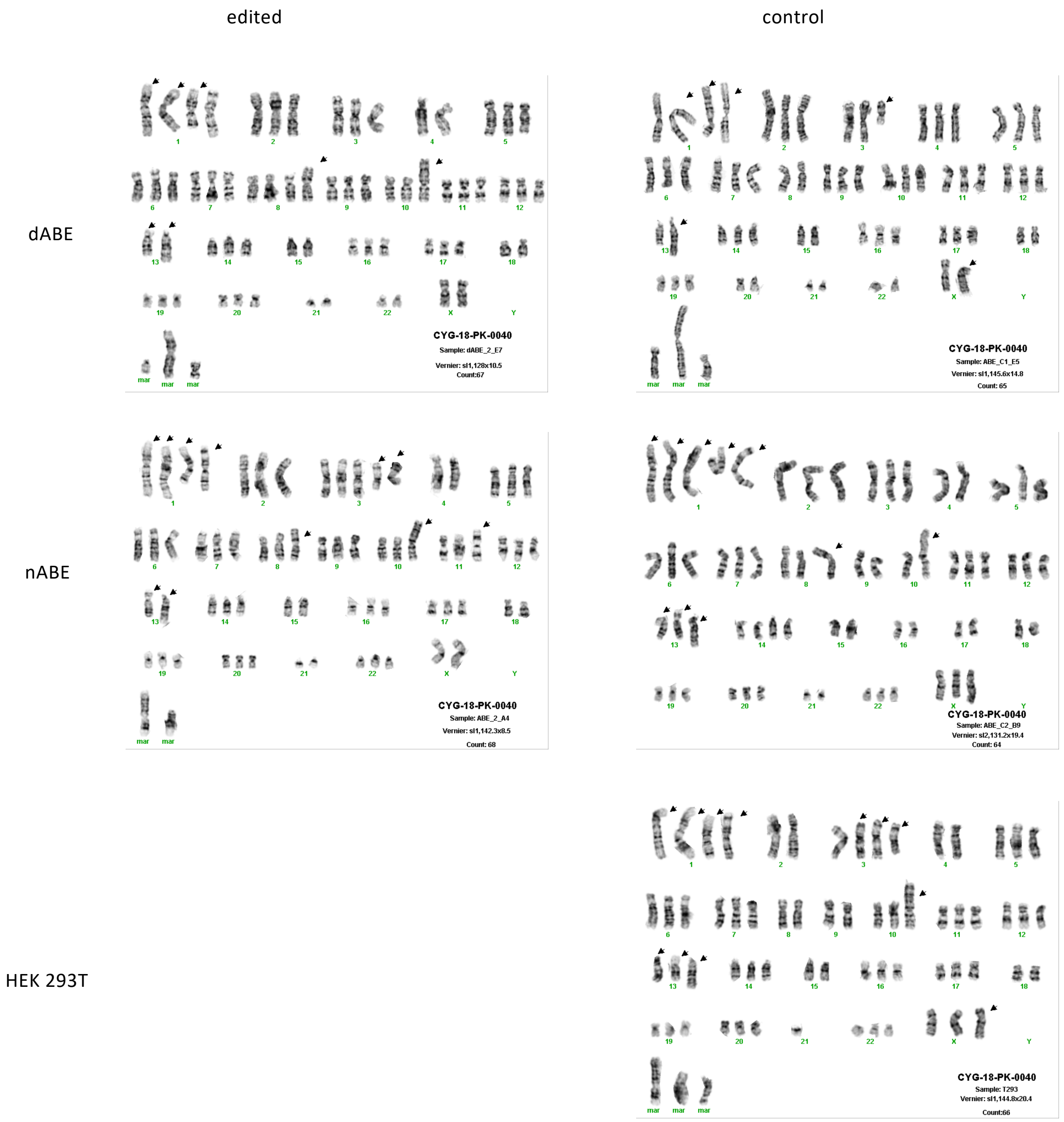

Figure S17 | Karyotype analysis after ABE editing. (A) Karyotype chromosome presentations for each of the genome edited clones and controls. The white arrows (hollow arrows) indicate non-clonal abnormalities and black arrows (solid arrows) indicate clonal abnormalities

A)

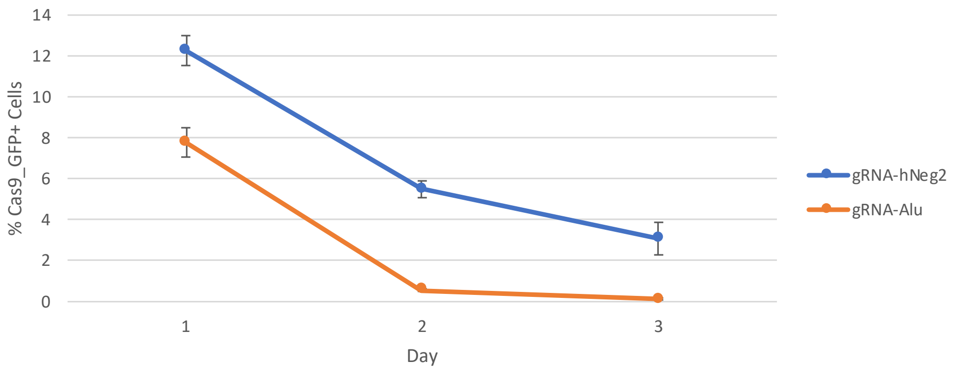

Figure S18 | TE gRNAs are highly toxic in human iPSCs (A) Percentage Cas9_GFP+ cells over time after transfection with TE or control gRNAs and Cas9.

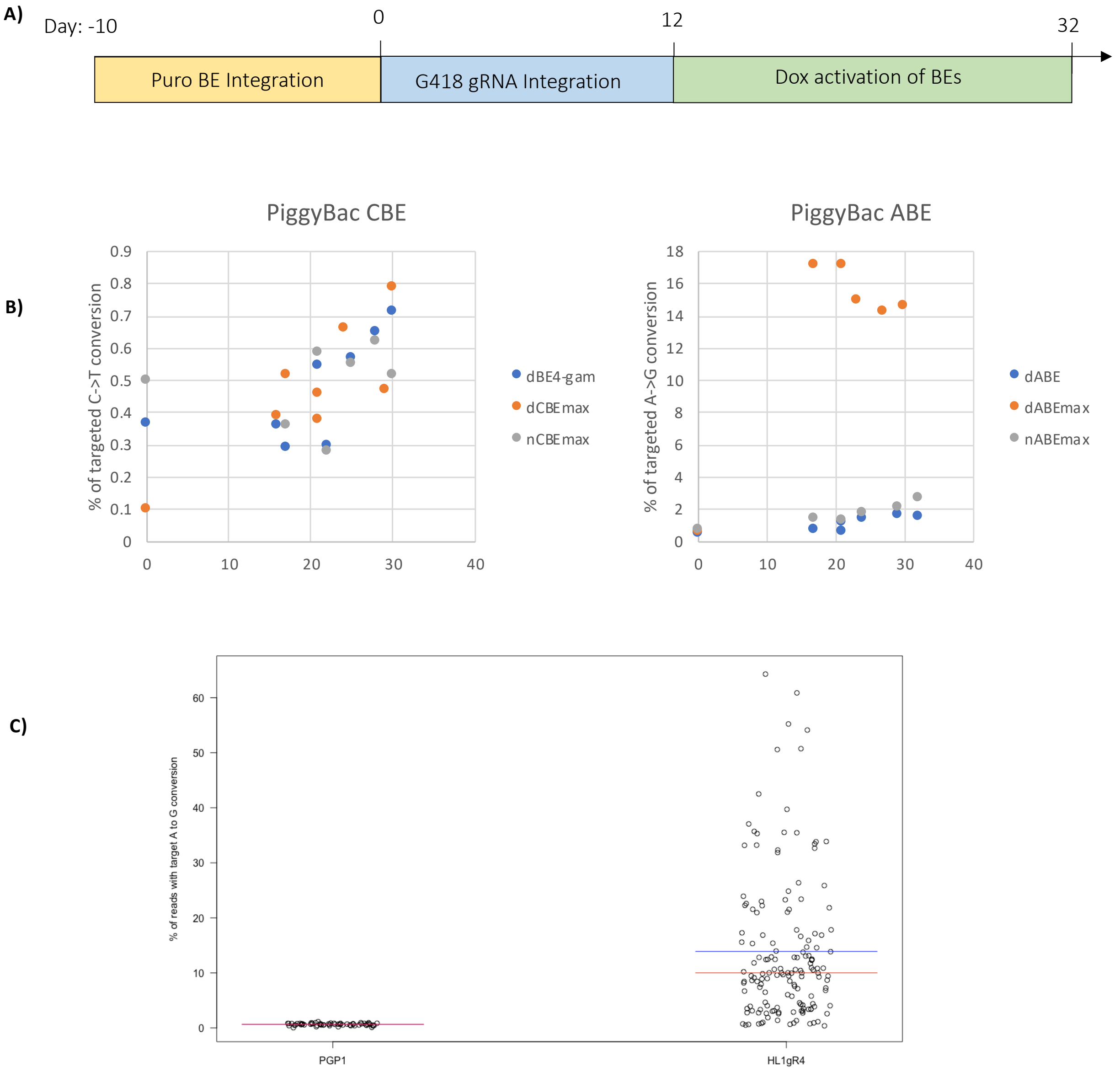

Figure S19| PiggyBac integration and extended editing of LINE-1 over time (A) Experimental overview of the genomic integration of BEs and HL1gR4 into PGP1 iPSCs using the Piggybac transposon and dual drug selection. At day 12 dox was added to the media to begin base editing at LINE-1 over time. **(B)** Editing time course of BEs in PGP1. **(C)** Single cell detection of LINE-1 mutation frequency using dABEmax and HL1gR4.

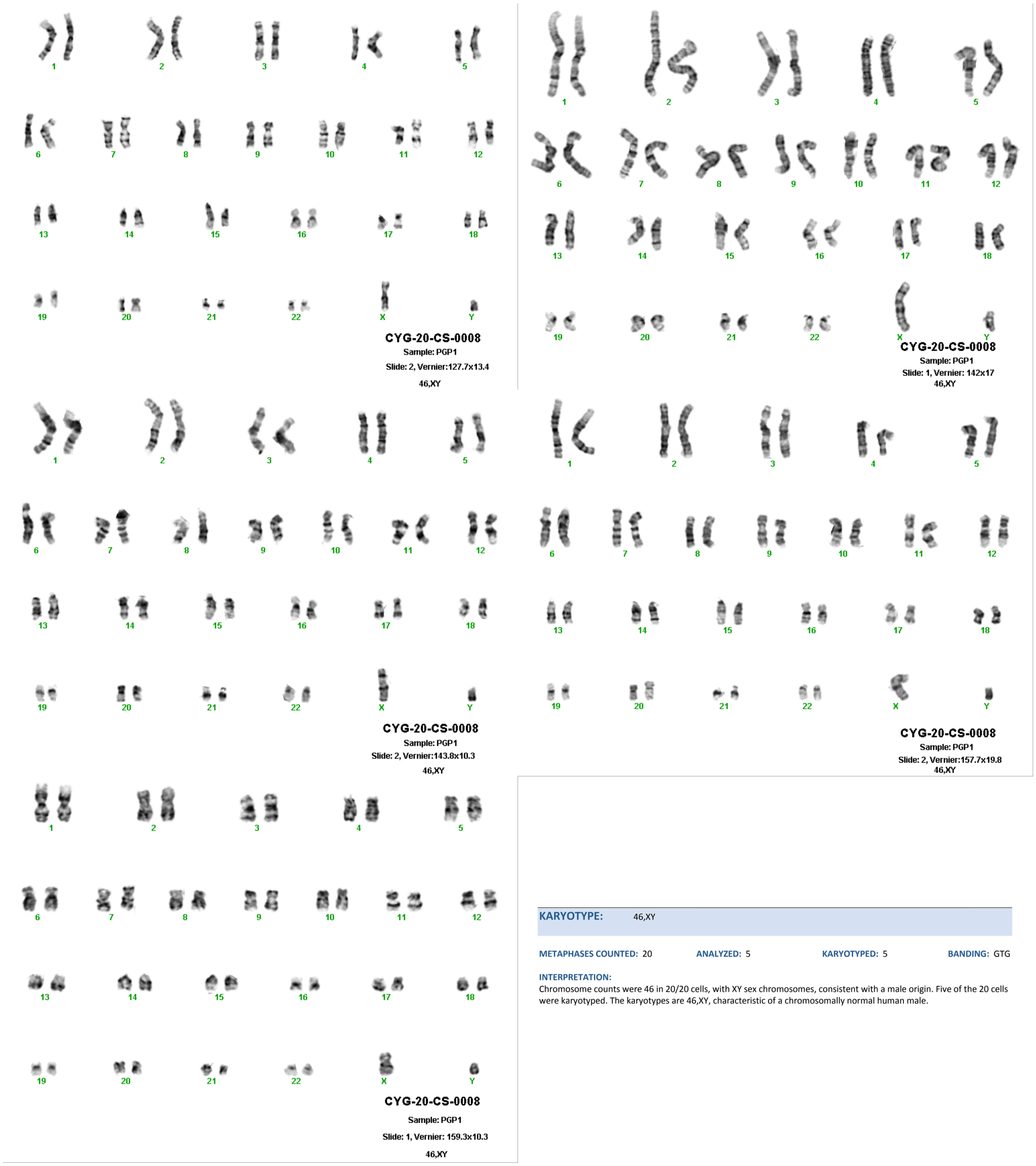

Figure S20 | Karyotype analysis of PGP1. 5 representative karyotype chromosome presentations for PGP1 iPSCs.

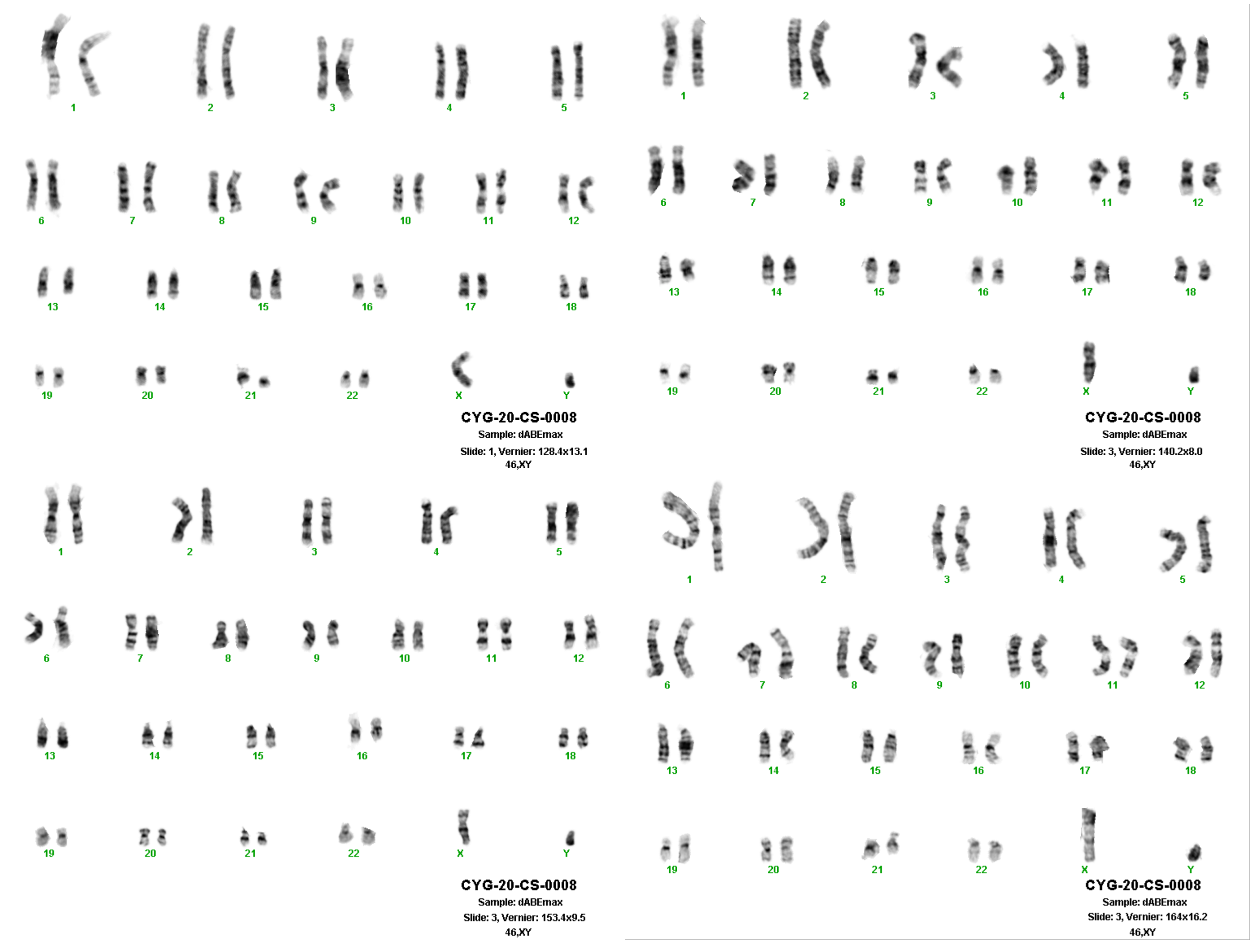

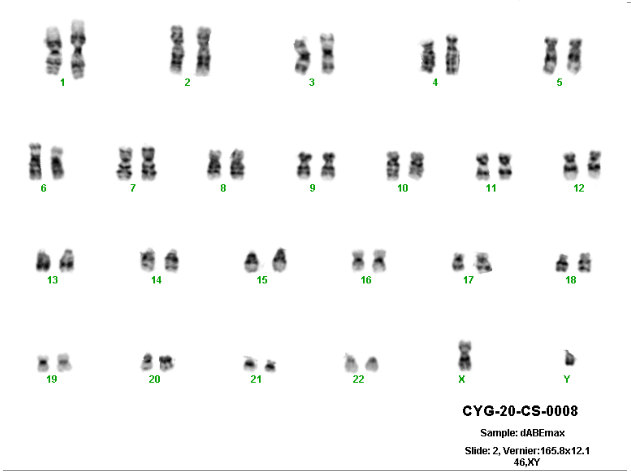

KARYOTYPE: 46,XY

METAPHASES COUNTED: 20 **ANALYZED:** 5 **KARYOTYPED:** 5 **BANDING:** GTG

INTERPRETATION:
Chromosome counts were 46 in 20/20 cells, with XY sex chromosomes, consistent with a male origin. Five of the 20 cells were karyotyped. The karyotypes are 46,XY, characteristic of a chromosomally normal human male.

Figure S21 | Karyotype analysis of PGP1 PB dABEmax. 5 representative karyotype chromosome presentations for PGP1 PB dABEmax iPSCs.

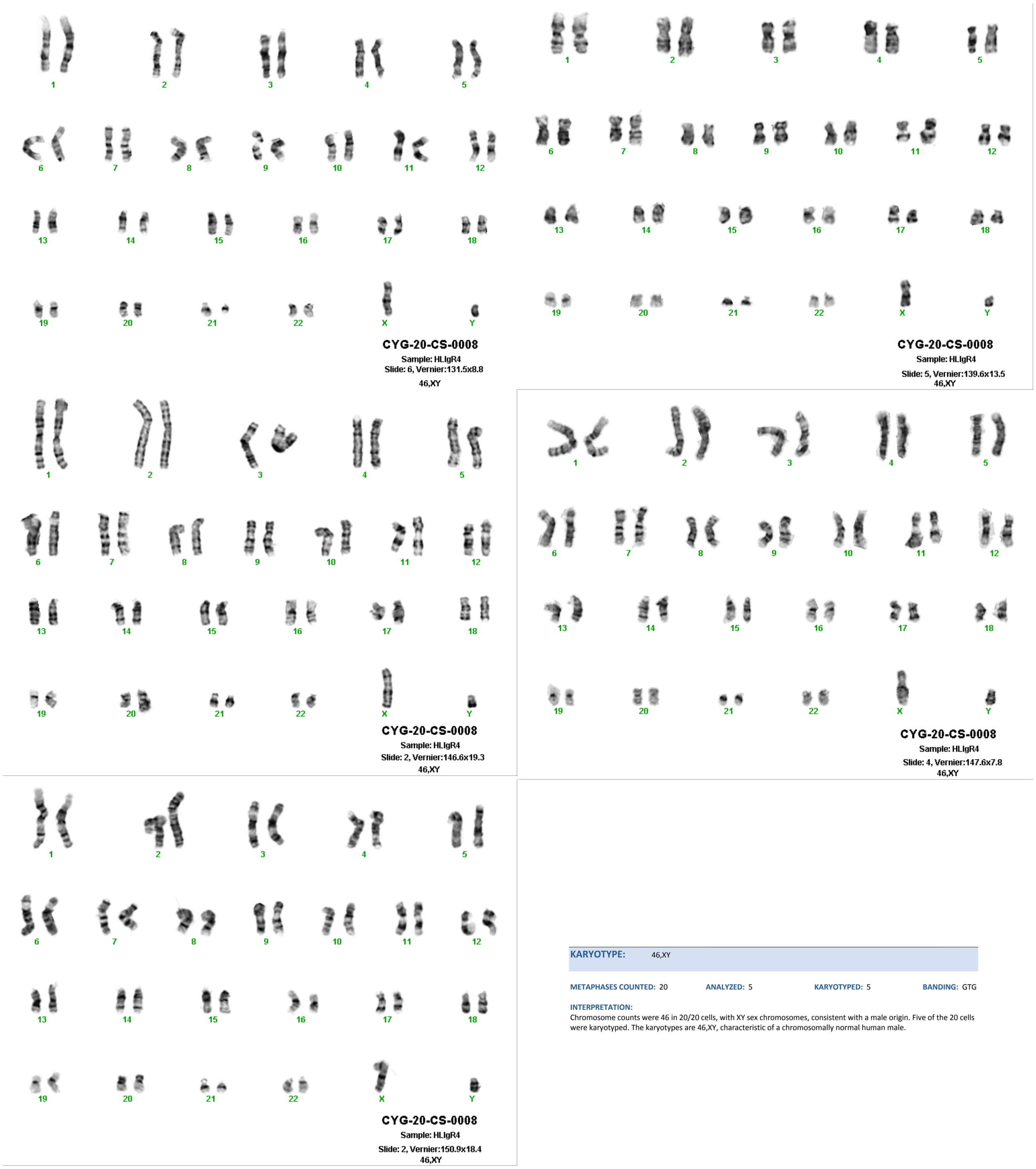

Figure S22 | Karyotype analysis of PGP1 PB dABEmax HL1gR4 edited. 5 representative karyotype chromosome presentations for PGP1 PB dABE PB HL1gR4 iPSCs with an average LINE-1 A->G conversion of 13.9%.

SUPPLEMENTAL TABLES

	C-deaminase	A-deaminase	UGI	Nick	Mu gam
dCBE1[1]	X				
dCBE2[1]	X		X		
nCBE3[1]	X		X	X	
nCBE4[2]	X		X2	X	
nCBE4-gam[2]	X		X2	X	X
dCBE4*	X		X2		
dCBE4-gam*	X		X2		X
nABE[3]		X		X	
dABE*		X			

*Synthesized and tested in this study

[1]Komor et al. (2016) *Nature* **533**(7603):420-4

[2]Komor et al. (2017) *Sci Adv* **3**(8):eaao4774

[3]Guadelli et al. (2017) *Nature* **551**:464-71

Table S1: Evolution of base editors

Manuscript name	Addgene name	Addgene #
pSB700	pSB700	64046
pSB700_mCherry	-	-
pSB700_Puro	-	-
SaCas9_gRNA	BPK2660	70709
pCas9_GFP	pCas9_GFP	44719
hCas9	hCas9	41815
nCBE2	pCMV_BE2	73020
nCBE3	pCMV_BE3	73021
nCBE4	BE4	100802
nCBE4-gam	BE4-gam	100806
nABE	pCMV_ABE7.10	102919
SaCas9	pX600-AAV-CMV::NLS-SaCas9-NLS-3xHA-bGHpA	61592
Sa-nCBE4-gam	SaBE4-gam	100809
SaKKH-nCBE4	pJL-SaKKH-BE3	85170
dCBE4	-	
dCBE4-gam	dCBE4-gam	
dABE	dABE	
nCBEmax	pCMV_AncBE4max	112094
dCBEmax		
nABEmax	pCMV_ABEmax	112095
dABEmax		

Table S2: List of DNA editors

```
ILMN – F:   5'– CTTTCCCTACACGACGCTCTTCCGATCT –3'
ILMN – R:   5'– GGAGTTCAGACGTGTGCTCTTCCGATCT –3'
```

gRNA Name	PrimerF	PrimerR
S1	TCAAGATGGCTGACAAAG	GCACCAGAGTCTCCGCTTTA
HL1 gR1	AGACTCCCACACATTAATAATGGG	TGATTTGGGGTGGAGAGTTCTG
HL1 gR2	AGTGCAATCAAACTAGAACTCAGG	CCCTCTACACACTGCTTTGAATG
HL1 gR3	AGTGCAATCAAACTAGAACTCAGG	CCCTCTACACACTGCTTTGAATG
HL1 gR4	AAGAGTCCAGGACCAGATGGAT	CCCGGCTTTGGTATCAGAATG
HL1 gR5	CTTATCCACCATGATCAAGTGGG	CTGCATCTATTGAGATAATCATGTGG
HL1 gR6	GTTCTGGCCAGGGCAATCAG	CCTGAGACTTTGCTGAAGTTGC
HL1 gR46	AACTGCAAGGCGGCAACGAG	AGAGGTGGAGCCTACAGAGG
EN gR1	CCAATACAGGAGCACCCAGATT	TGATTTGGGGTGGAGAGTTC
EN gR9	CAGAACTCTCCACCCCAAAT	CCTGAGTTCTAGTTTGATTG
RT gR1	CCACATGATTATCTCAATAG	GAGGGCATCCCTGTCTTGTG
RT gR3	GCAACTTCAGCAAAGTCTCA	GTAGTTCTCCTTGAAGAGGTCC
EN (dual gRNA)	CCAATACAGGAGCACCCAGATT	CCCTCTACACACTGCTTTGAATG
RT (dual gRNA)	CCACATGATTATCTCAATAG	GTAGTTCTCCTTGAAGAGGTCC
ENRT (dual gRNA)	CAGAACTCTCCACCCCAAATC	CCCGGCTTTGGTATCAGAATG
shEN (dual gRNA)	CAGAACTCTCCACCCCAAATC	CCCTCTACACACTGCTTTGAATG
HERV env11	AATACCACCCTCACTGGGCT	CAGATTGGAAACAAGAGGTCC

Table S3: NGS primers list

Name	gRNA	PAM	Cas9 species
Non human	GAGACGATTAATGCGTCTCG	NGG	SpCas9
S1	GATGACAGGCAGGGGCACCG	CGG	SpCas9
HL1 gR1	AACGAGACAGAAAGTCAACA	AGG	SpCas9
HL1 gR2	TCAGTTTCCATGTAGTTGAG	CGG	SpCas9
HL1 gR3	TATGTACCCAGTAGTCATTC	AGG	SpCas9
HL1 gR4	ATTCTACCAGAGGTACAAGG	AGG	SpCas9
HL1 gR5	TTGAACCAGCCTTGCATCCC	AGG	SpCas9
HL1 gR6	GGGTATTCAATTAGGAAAAG	AGG	SpCas9
EN gR1	GACTCCCACACATTAATAAT	GGG	SpCas9
HL1 gR46	GCTTAGGTAAACAAAGCAGC		SpCas9
EN gR9	ATTTTGGAATAGGTGTGGTG	TGG	SpCas9
RT gR1	ATTCAGTATGATATTGGCTG	TGG	SpCas9
RT gR3	CCTAGGAATCCAACTTACAA	GGG	SpCas9
Z8 gR2	AAAAAGAGTCCAGGACCAGA	TGG	SpCas9
Alu	CAGGCGTGAGCCACCGCGCC	CGG	SpCas9
Sa Non human	GAGACGATTAATGCGTCTCG	NGG	SaCas9
HERV env11	GAGGCACATCCAACAGTTAG	TAGGG	SaCas9
ABEgR1	GAACACAAAGCATAGACTGC	CGG	SpCas9
AATF-B4	GATGACGCGGAAGACTCCCA	TGG	SpCas9
ACTL7B-B9	CCGTGTGGACCGGCGGTTCC	TGG	SpCas9
ACSBG2-C10	AGGGACCACAGGCATACCCA	AGG	SpCas9
ABCG4-B3	GAAGCGTCTGGCCATCGCCC	CGG	SpCas9
ABT1-C9	CTACACCAAGGACTACACCG	AGG	SpCas9
ACTC1-B1	CTGGTGAGTGTGTGTGTCTC	AGG	SpCas9
A1CF-C4	GGTGCAGCATCCCAACCAGG	CGG	SpCas9

Table S4: gRNAs used in this study

# of mismatches	reads	total reads	percentage
0	22780	35520	64.1%
1	6546	35520	18.4%
2	1131	35520	3.2%
3	148	35520	0.4%
4	63	35520	0.2%
5	2	35520	0.0%
6	26	35520	0.1%
7	1	35520	0.0%
8	1	35520	0.0%
9	1	35520	0.0%
>9	4600	35520	13.0%

Table S5: LINE-1 subfamily analysis and matches to HL1gR4

	BE4_2_A11	BE4_C2_A7	dBE4_3_C6	dBE4_C1_B2	ABE_2_A4	ABE_C2_B9	dABE_2_E7	dABE_C1_E2	293T9(CYG-18-PK-0040)
-X				x	x		x	x	
add(X)(q28)	x	x	x		x	x			x
der(X)add(X)(p11.2)add(X)(q28)								x	
add(1)(p36.1)	x	x	x	x	x	x	x		x
add(1)(q42)	xx	xx	xx	xx	xx	xx	xx		xx
del(1)(q31)	x	x	x	x		x	x		x
i(1)(p10)								x	
add(1)(q21)								x	
-2									
add(3)(p13)								x	
add(3)(p24)			xx						x
del(3)(p22)	x				x				
add(3)(q12)			x	x					x
del(3)(q22)	x				x	x		x	
add(4)(p15)	x								
del(4)(q31)	x								
-4		x			x	x	x		x
add(8)(p21)	x	x	x	x	x	x	x		x
-9						x			
add(10)(p11)									
add(10)(p13)	x	x	x	x	x	x	x		x
add(11)(p15)			x		x				
add(13)(p11)	xx	xx	xx	xx	xx	xx	xx		xx
add(13)(q34)	x	x	x	x	x	x	x		x
-13									
add(14)(p11.2)			x	x				x	
-15	x	x	x	x	x	x	x		x
add(15)(p11.2)		x							
-18	x	x	x	x	x	x	x		x
-21	x	x		x	x	x	x		x
-22							x		
i(21)(q10)			x						
mar	x-xx	x-xx	x-xxx	x-xx	xx-xxx	x-xx	x-xxx	x-xxx	x-xxxx

Table S6: Karyotype chromosomal abnormality list

SUPPLEMENTARY RESULTS

Single cell analysis of LINE-1 dual gRNA disrupted cells

The PCR amplicons of dual gRNA combinations from the previous experiments were too large to include both the mutated and full-length bands together for Illumina NGS. To overcome this, a shorter pair of LINE-1 targeting gRNAs, called short EN (shEN), was used that permits both regions to be sequenced together. 293Ts were transfected with pCas9_GFP and the shEN gRNA pair (ENgR9 and HL1gR3) in the pSB700mCherry gRNA expression vector (*fig. S4A*). GFP and mCherry double-positive single cells were FACS-sorted into gDNA extraction solution. 303 individual cells were screened after FACS sorting and LINE-1 NGS analysis. Of those wells with an amplicon, 83.24% had a visually detectable deletion band with a range of intensities from barely observable to stronger than the wild type non-mutated band (*fig. S4B*). Bulk-transfected cells had a dual gRNA deletion frequency of 2.7%, the FACS-enriched double-positive cell population was edited at 11.19%, and the mean editing of single-cell-derived amplicons was ~50.17% (*fig. S4C*). The editing frequency appears to be bimodal as previously reported in the set of PERV editing papers, with experiments first in transformed cells[8] – achieving 62 indels – and then later in healthy born piglets[9] – with all 25 PERVs knocked-out. At first it seems contradictory that the population bulk gDNA editing efficiency is 11.19% and the single cell average is 50.1% but this assumes that each single cell had a full nuclear genome. The highest edited samples most likely already had degraded their genomes due to the thousands of concurrent cuts to every chromosome received; thus, each single cell observed was contributing unequally to the bulk population.

nBE activity confirmed at LINE-1 for both nABE and nCBE

We tested the efficiency of deamination at LINE-1 using base editors. We designed and tested LINE-1 targeting gRNAs (HL1gR1-6 [*table S1*]) that generate a STOP codon early in ORF-2 using C->T deamination. HEK 293T cells were transfected with nCBE3 and gRNAs individually. Deamination events were detected at each of the six gRNA target loci above the background levels of editing observed (~0.05% – 0.67%) in mock transfected populations of cells (*fig. S5A*). These same CBE gRNAs were also compatible with ABEs as they contain at least one adenine within their target window. Base editing with nCBE4-gam and nABE was detected in the population in 4/5 gRNAs for CBE (*fig. S5B*) and 4/5 gRNAs for ABE (*fig. S5C*). nABE had the highest editing efficiency using HL1gR6 at 4.94% or ~1290 loci genome wide. HL1gR4 was chosen as the best target for future studies as its signal to background error ratio was the lowest of all the LINE-1 amplicons/gRNAs tested, and the HL1gR4 was among the most efficient. The HL1gR4 target also contains three C's within its target window that are all efficiently co-edited as a clear watermark of mutation.

RNA-seq in LINE-1 KO clones

We did not observe downregulation of LINE-1 RNA expression levels in edited clones. In *Fig. S8B* the number of RNA reads obtained through the standard deamination analysis pipeline, averaged over the 20 nt protospacer sequence and normalized the read counts by dividing by the size of their respective libraries, are displayed. A list of predicted differentially expressed genes in the edited clones compared to the wild type is found in supplementary data S1, and numbers of up and down regulated genes is found in *fig. S8C*. Multidimensional scaling of the gene expression data (*Fig. S8D*), where the distance between the samples corresponds to leading log-fold-changes between the RNA samples, shows a clear separation between the wild type and the three edited

samples. Since the wild type control samples, however, did not undergo a comparable procedure of transfection and cell sorting, we cannot conclude that the observed differences in gene expression are due to LINE-1 editing.

DATA AND MATERIALS AVAILABILITY

Key plasmids developed during this study have been submitted to Addgene: pSB700_HL1 gR4 (# 124450), dABE (# 124447) and dCBE4-gam (# 124449). All NGS data used for the figures and supplementary figures have been made available at SRA BioProject Accession #PRJNA515875, #PRJNA518077, and #PRJNA561375 for 293T, PGP1 and WGS respectively.

SUPPLEMENTARY REFERENCES

1. Penzkofer, T. *et al.* L1Base 2: more retrotransposition-active LINE-1s, more mammalian genomes. *Nucleic Acids Res* **45**, D68–D73 (2017).
2. Grandi, N., Cadeddu, M., Blomberg, J. & Tramontano, E. Contribution of type W human endogenous retroviruses to the human genome: characterization of HERV-W proviral insertions and processed pseudogenes. *Retrovirology* **13**, 67 (2016).
3. Chavez, A. *et al.* Highly efficient Cas9-mediated transcriptional programming. *Nat Meth* **12**, 326–328 (2015).
4. Kleinstiver, B. P. *et al.* Broadening the targeting range of Staphylococcus aureus CRISPR-Cas9 by modifying PAM recognition. *Nat. Biotechnol.* **33**, 1293–1298 (2015).
5. Byrne, S. M. & Church, G. M. Crispr-mediated Gene Targeting of Human Induced Pluripotent Stem Cells. *Curr Protoc Stem Cell Biol* **35**, 5A.8.1-22 (2015).
6. Langmead, B. & Salzberg, S. L. Fast gapped-read alignment with Bowtie 2. *Nature Methods* **9**, 357–359 (2012).
7. Li, H. *et al.* The Sequence Alignment/Map format and SAMtools. *Bioinformatics* **25**, 2078–2079 (2009).

8. Yang, L. *et al.* Genome-wide inactivation of porcine endogenous retroviruses (PERVs). *Science* **350**, 1101–1104 (2015).
9. Niu, D. *et al.* Inactivation of porcine endogenous retrovirus in pigs using CRISPR-Cas9. *Science* (2017) doi:10.1126/science.aan4187.
